# Dipeptidyl peptidase 4 promotes peritoneal fibrosis and its inhibitions prevent failure of peritoneal dialysis

**DOI:** 10.1038/s42003-021-01652-x

**Published:** 2021-01-29

**Authors:** Yi-Chen Li, Pei-Hsun Sung, Yao-Hsu Yang, John Y. Chiang, Hon-Kan Yip, Chih‐Chao Yang

**Affiliations:** 1 Division of Cardiology, Department of Internal Medicine, Kaohsiung Chang Gung Memorial Hospital and Chang Gung University College of Medicine, Kaohsiung, Taiwan; 2 Center for Shockwave Medicine and Tissue Engineering, Kaohsiung Chang Gung Memorial Hospital, Kaohsiung, Taiwan; 3 Institute for Translational Research in Biomedicine, Kaohsiung Chang Gung Memorial Hospital, Kaohsiung, Taiwan; 4 Department of Traditional Chinese Medicine, Chang Gung Memorial Hospital, Chiayi Branch, Putzu, Taiwan; 5 Health Information and Epidemiology Laboratory of Chang Gung Memorial Hospital, Chiayi Branch, Putzu, Taiwan; 6 School of Medicine, Chang Gung University, Taoyuan, Taiwan; 7 Institute of Occupational Medicine and Industrial Hygiene, National Taiwan University College of Public Health, Taipei, Taiwan; 8 Department of Computer Science and Engineering, National Sun Yat-sen University, Kaohsiung, Taiwan; 9 Department of Healthcare Administration and Medical Informatics, Kaohsiung Medical University, Kaohsiung, Taiwan; 10 Department of Nursing, Asia University, Taichung, Taiwan; 11 Department of Medical Research, China Medical University Hospital, China Medical University, Taichung, Taiwan; 12 Division of Cardiology, Department of Internal Medicine, Xiamen Chang Gung Hospital, Xiamen, Fujian China; 13 Division of Nephrology, Department of Internal Medicine, Kaohsiung Chang Gung Memorial Hospital and Chang Gung University College of Medicine, Kaohsiung, Taiwan

**Keywords:** Mechanisms of disease, Peritoneal dialysis, End-stage renal disease, Translational research

## Abstract

Peritoneal dialysis (PD) possesses multiple advantages for end stage renal disease. However, long-term PD triggers peritoneal fibrosis (PF). From the nationwide analysis of diabetic PD patients (*n* = 19,828), we identified the incidence of PD failure was significantly lower in diabetic patients treated with dipeptidyl peptidase 4 (DPP4) inhibitors. Experimental study further showed high concentration of glucose remarkably enhanced DPP4 to promote epithelial-mesenchymal transition (EMT) in the mesothelial cells. In chlorhexidine gluconate (CG)-induced PF model of rats, DPP4 expression was enriched at thickening peritoneum. Moreover, as to CG-induced PF model, DPP4 deficiency (F344/DuCrlCrlj strain), sitagliptin and exendin-4 treatments significantly inhibited DPP4 to reverse the EMT process, angiogenesis, oxidative stress, and inflammation, resulting in the protection from PF, preservation of peritoneum and the corresponding functional integrity. Furthermore, DPP4 activity was significantly correlated with peritoneal dysfunction. Taken together, DPP4 caused peritoneal dysfunction/PF, whereas inhibition of DPP4 protected the PD patients against PD failure.

## Abstract (web summary)

Li et al. show that dipeptidyl peptidase 4 (DPP4) promotes peritoneal fibrosis whereas the inhibition of DPP4 protects the patients from failure of peritoneal dialysis. This study provides mechanistic insights into the effects of DPP4 on peritoneal fibrosis and the translational potential of these effects.

## Introduction

The incidence and prevalence rates of end-stage renal disease (ESRD) keep on increasing and have been emerged as a global health burden^1^. Studies have further demonstrated the overall mortality rate of ESRD patients is unacceptably high with an estimation of up to 15–30% per year^2^. Among the ESRD patients, renal transplantation has been reported to possess the better long-term survival outcome as compared to that of peritoneal dialysis (PD) or conventional hemodialysis (HD) patients. However, not every ESRD patient has the chance or as a candidate for receiving renal transplantation^3^. Available data have reported that PD is currently utilized by ~270,000 ESRD patients worldwide, representing ~12% of the total dialysis population^1,4,5^. Compared with conventional HD patients, PD patients have a higher survival rate during the first 2 years of dialysis treatment and a better quality of life^6^. However, 20% of PD patients are finally turned to receive conventional HD (defined as PD failure) because of loss of functional integrity of peritoneal ultrafiltration affected by several unfavorable factors^7,8^. In fact, the success of PD is mainly dependent on the structural and functional integrity of the peritoneal membrane^9^. The peritoneal membrane, a rather simple histological ultrastructure, contains a superficial epithelial-like cell layer (i.e., the mesothelium) attached to a basement membrane. Beneath the basement membrane is a submesothelial layer which consists of the connective tissue, fibroblasts, and blood vessels^10,11^.

During PD, the peritoneal membrane acts as an endogenous dialyzing membrane by the permeability of peritoneal capillaries. Across this membrane, waste products diffuse into the dialysate that is usually glucose-containing fluid with solute transport capacity. Commercialized PD solutions are composed of acidic pH, high-glucose concentration, and high osmolality, resulting in generation of glucose degradation products (i.e., a by-product during standard sterilization), and in further jeopardizing the peritoneal architectural integrity^12^. In this chemically gradient situation, the excess body fluid is removed by osmosis^13^. Nevertheless, the phenomena of angiogenesis, vascular degeneration, and peritoneal fibrosis (PF), i.e., so-called peritoneal ultrastructural and functional damages, frequently induced by long-term exposure to PD solution, are closely associated with increased solute transport and decreased ultrafiltration capacity in ~50% of all PD patients^14–16^.

Dipeptidyl peptidase IV (DPP4) is a 110-kDa type II integral membrane glycoprotein with serine peptidase activity to degrade incretins such as glucagon-like peptide-1 (GLP-1), and is widely expressed on the surface of various cell types, endothelial cells, kidney epithelial cells, immune cells, and mesothelial cells^17–21^. There are two kinds of incretin-based therapies for the treatment of type 2 diabetes, i.e., DPP4 inhibitors and glucagon-like peptide-1 receptor agonist^22^. The DPP4 inhibitor has been recommended as one of the most preferable option among oral hypoglycemic agents^23^. Of particular importance is that DPP4 inhibitor has been proved to exert cytoprotective effects, such as anti-inflammatory^24,25^, antifibrotic^26^, and antiapoptotic effects^27^. Thus, DPP4 inhibitor has pleiotropic effects beyond hypoglycemic effect. Basic research has recently demonstrated that an interaction between DPP4 and integrin β1 would initially elicit the endothelial cells into endothelial–mesenchymal transition (EndMT) (i.e., a morphological and functional changes) and finally into fibrosis through activating Smad-3 pathway in kidney injury^28^. On the other hand, inhibition of DPP4 activity possesses antifibrotic effects and provides renoprotection^29^.

Interestingly, our previous study demonstrated that DPP4 enzyme deficiency protected kidney against acute ischemia-reperfusion injury^25^. In addition, the other report showed the prevention of DPP4 inhibitor in PF of mice, but it did not explore DPP4 expression or activity during PF^30^. So far, it has still lacked a direct evidence to address the pathogenic roles and molecular mechanisms of DPP4 involving in peritoneal pathogenesis. Furthermore, the effective protection of DPP4 inhibitors in clinical PD patients is unclear. Therefore, we first analyzed a 17-year observational data from Taiwan National Health Insurance Research Database to explore whether DPP4 inhibitor could inhibit chronic PD-induced peritoneal fibrosis and further reduce the rate of PD failure. Since the roles of DPP4 in the peritoneal pathogenesis have been not yet clearly established, we subsequently examined the cellular and molecular mechanisms of DPP4 on peritoneal fibrosis and the role of incretin-based therapies on protecting the peritoneum against fibrosis by using in vitro and peritoneal-fibrosis animal model.

## Results

### DPP4 inhibitors reduced the incidence of PD transition to HD

First, we examined the nationwide registered data and studied the incidence of PD transition to HD (i.e., defined as PD failure) among ESRD patients who had regularly received different kinds of DPP4 inhibitor for controlling type 2 diabetes. Table 1 shows the prevalence of user of DPP4 inhibitor was about 10% of patients with ESRD, with a similar prevalence of utilizing this medication among patients in PD, HD, and RT (Table 1). Of note, the rate of conversion of PD to HD was significantly lower in diabetic patients than in those without receiving DPP4 inhibitors (Table 2).

**Table 1:** Percentage of users and nonusers of DPP4i among diabetic ESRD patients with different dialysis modalities.

	Users of DPP4i		Nonusers of DPP4i	
	*N*	%	*N*	%
All cases of ESRD (*N* = 174686)	16,048	9.2	158,638	90.8
PD (*N* = 19828)	2150	10.8	17,678	89.2
HD (*N* = 171266)	15,634	9.1	155,632	90.9
RT (*N* = 6787)	771	11.4	6016	88.6

*DPP4i* dipeptidyl peptidase-4 inhibitor, *ESRD* end-stage renal disease*, PD* peritoneal dialysis, *HD* hemodialysis, *RT* renal transplantation, *N* number, % percentage.

**Table 2:** Comparison of rate of PD transition to HD between groups.

	Users of DPP4i (*N* = 541)		Nonusers of DPP4i (*N* = 6449)		
PD transition to HD	*N*	%	*N*	%	*P*-value
Yes	177	32.7	3393	52.6	<0.0001
No	364	67.3	3056	47.4	

*DPP4i* dipeptidyl peptidase-4 inhibitor, *ESRD* end-stage renal disease, *PD* peritoneal dialysis, *HD* hemodialysis, *RT* renal transplantation, *N* number, % percentage.

Among all types of antidiabetic drugs listed in Table 3, those diabetic ESRD patients receiving PD and DPP4 inhibitor had the lowest rate of PD transition to HD (Table 3). On the contrary, about one-half of ESRD patients who were administered with other antidiabetic drugs suffered from PD catheter failure at the end of study, implying DPP4 inhibitor offered a better protective effect against peritoneal sclerosis or fibrosis compared to other non-DPP4 inhibitors antidiabetic drugs (Tables 2 and 3).

**Table 3:** Rate of PD transition to HD among subgroups of antidiabetic drugs.

	Number and percentage of antidiabetic drugs used in ESRD on PD (*N* = 6990)		Rate of PD transition to HD (*N* = 3570)		Comparison to DPP4 inhibitor
Antidiabetic drugs	*N*	%	*N*	%	*P-*value
Insulin	1813	25.9	916	50.5	<0.0001
Sulfonylurea (SFU)	938	13.4	475	50.6	<0.0001
Meglitinide (GLN)	825	11.8	397	48.1	<0.0001
TZD	434	6.2	220	50.7	<0.0001
AGI	332	4.8	166	50.0	<0.0001
DPP4 inhibitor	541	7.7	177	32.7	–

*PD* peritoneal dialysis, *HD* emodialysis, *ESRD* end-stage renal disease, *N* number, % percentage.

Sulfonylureas: Gliclazide, Glimepiride, Glyburide, etc.; Meglitinides: Nateglinide, Repaglinide; Thiazolidinediones (TZD): Pioglitazone, Rosiglitazone; Alpha-glucosidases inhibitor (AGI): Acarbose; Dipeptidyl peptidase-4 (DPP4) inhibitors: Linagliptin, Saxagliptin, Sitagliptin, Alogliptin, Vildagliptin.

### Glucose exposure upregulated DPP4, promoted EMT process, and enhanced inflammation, respectively, via activation of SMAD3 and NFκB pathway in mesothelial cells

Previous reports have demonstrated that the majoring of ESRD patients’ peritoneal fibrosis was caused by the long-term exposure of PD solution, resulted in a histopathological phenomenon of mesothelial cells underwent epithelial–mesenchymal transition (EMT) process^31,32^. Accordingly, to further explore the role of DPP4 and potential mechanism in peritoneal fibrosis, we investigated the molecular changes of mesothelial cells on a stepwise increment of glucose exposure.

**Fig. 1:**
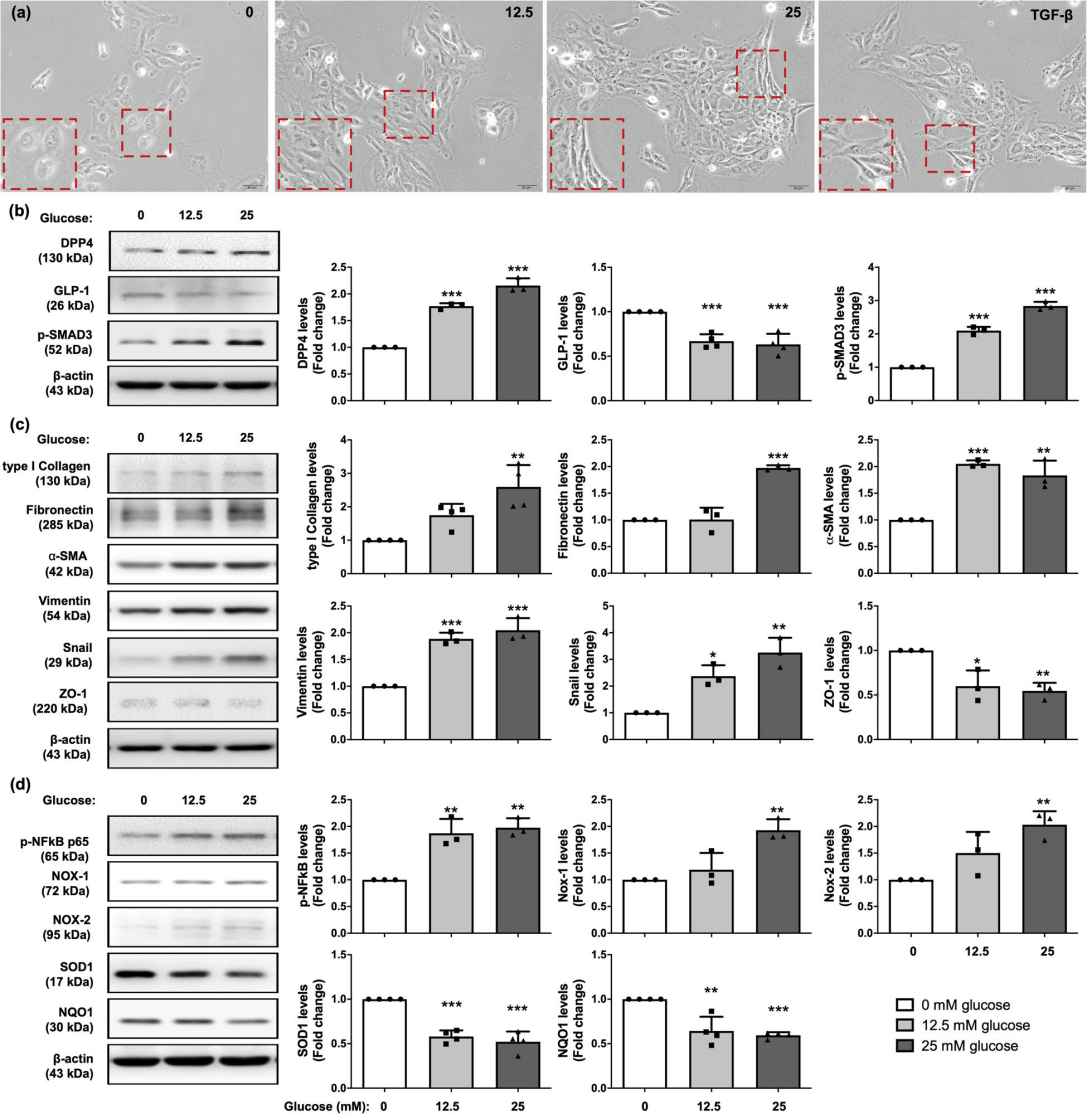
Glucose treatment induced increase of DPP4 levels, EMT process, oxidative stress generation, and inflammation in mesothelial cells. **a** Met-5A cells were exposed in 0, 12.5, 25 mM D-glucose, and 2 ng/ml TGF-β1 recombinant protein for 120 h and observed cell morphology by microcopy at 200×. The Met-5A cells were changed from cobble stone shapes to fibroblast-liked morphologies in glucose-dependent manner. TGF-β1 treatment was as a positive control of EMT. Scale bars: 50 μm. **b** Western blotting was performed to detect the protein levels of DPP4, p-SMAD3, and GLP-1. Quantitative results were shown in the right panel. The relative levels were normalized and compared by 0 mM glucose concentration. **c** Western blotting was performed to detect the protein levels of the mesenchymal markers (type I collagen, fibronectin, α-SMA, vimentin, Snail) and epithelial marker (ZO-1). **d** Glucose treatment enhanced phosphorylated NF-kB activation, elevated the levels of ROS generated enzyme (NOX-1, NOX-2) but declined antioxidant enzymes (SOD1, NQO1) to cause ROS accumulation detected by immunoblotting. Data represents the analysis of at least 3 independent experiments and shows mean ± SD. * indicates *p*-value <0.05; ** represents *p*-value <0.01; and *** is *p*-value <0.001.

Consistent with previous reports^31,32^, our results demonstrated that as glucose concentration increased, the morphology of mesothelial cells transformed from cobble stone shapes to fibroblast-liked structure in a glucose concentration-dependent manner (Fig. 1a). The western blot result showed that the protein level of DPP4 was significantly progressively increased with stepwise increased glucose concentration, whereas protein expression of GLP-1 exhibited an opposite pattern of DPP4 in the similar situation (Fig. 1b). In addition, glucose exposure activated p-SMAD3 (Fig. 1b), which in turn, upregulated the snail level, followed by increasing the mesenchymal markers (i.e., type I collagen, fibronectin, α-SMA, vimentin, Twist) but decreasing the epithelial marker (Zonula occludens-1, ZO-1). All of these findings shared glucose-dependent trends (Fig. 1c, Supplementary Fig. 1).

Furthermore, glucose exposure also enhanced NFκB pathway and generated oxidative stresses (NOX-1 and NOX-2) (Fig. 1d). In contrast, glucose exposure suppressed the enzymes of clearing oxidative stress such as SOD1 and NQO1 (i.e., antioxidant enzyme) (Fig. 1d). Our finding reveals high concentration of glucose exposure, a major component of PD solution, triggered DPP4 induction, underwent EMT process, and generated inflammation and oxidative stress.

### Sitagliptin and exendin-4 treatments suppressed glucose-induced DPP4 expression, EMT processing, inflammation, and oxidative stress in mesothelial cells

**Fig. 2:**
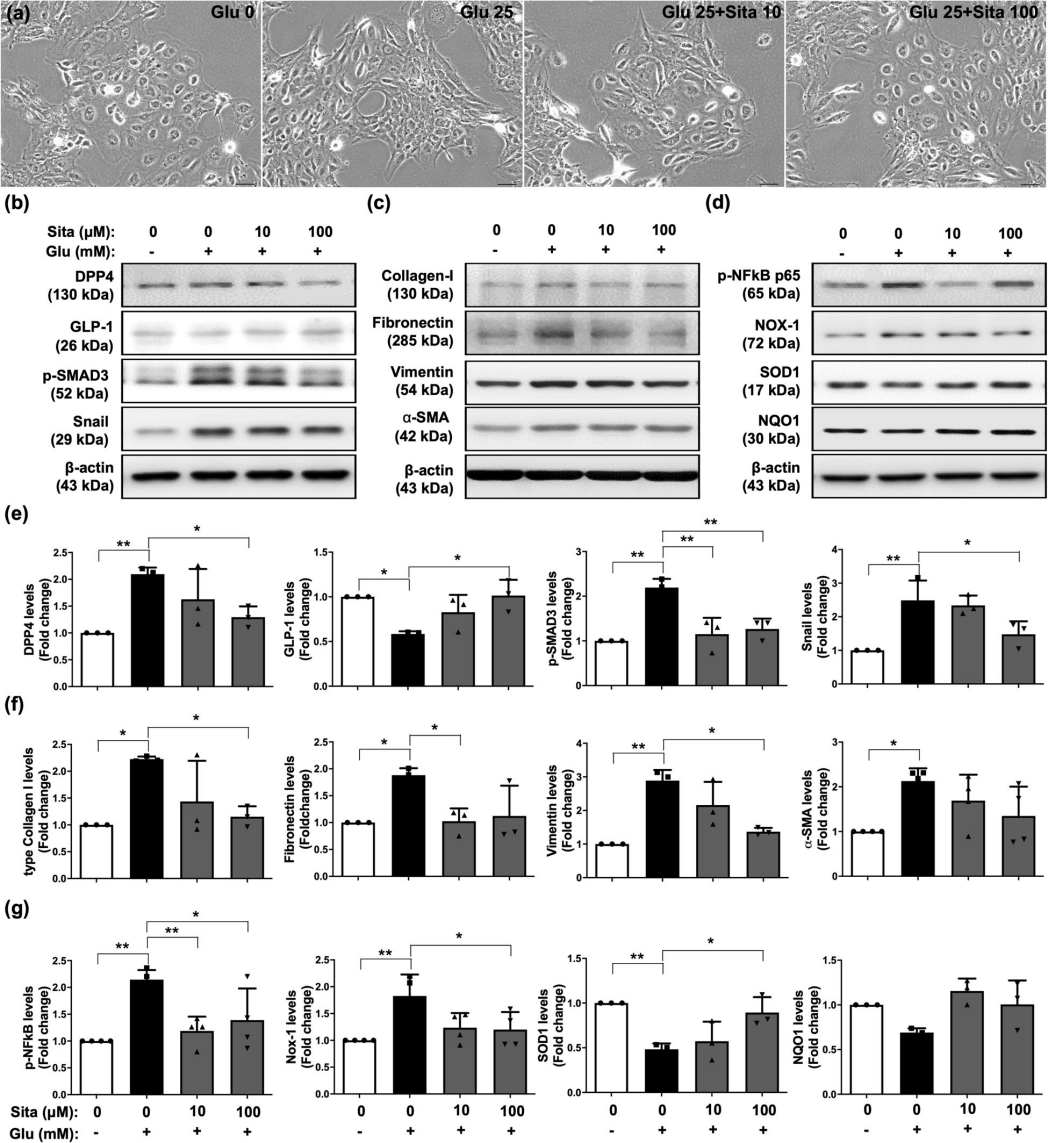
Sitagliptin suppressed glucose-inducing DPP4 increase, EMT process, inflammation, and oxidative stress in mesothelial cells. **a** Met-5A cells were induced with or without 25 mM D-glucose (Glu) exposure for 120 h. Under 25 mM D-glucose (Glu) induction, Met-5A cells were treated sitaglipin (Sita) with 0, 10, or 100 μM concentrations, and then observed cell morphology by microcopy at 200×. Scale bars: 50 μm. **b** Western blotting was performed to detect the protein levels of DPP4, GLP-1, p-SMAD3, and Snail. **c** Western blotting was performed to evaluate the protein levels of collagen-I (type I collagen), fibronectin, vimentin, and α-SMA. **d** Western blotting was performed to analyze the protein levels of p-NF-kB, NOX-1, SOD1, and NQO1. **e**–**g** Quantitative results of Fig. 2b, c, and d were shown, respectively. The relative levels were normalized and compared by 0 mM glucose concentration. Data represents the analysis of at least 3 independent experiments and shows mean ± SD. * indicates *p*-value < 0.05; ** represents *p*-value <0.01.

**Fig. 3:**
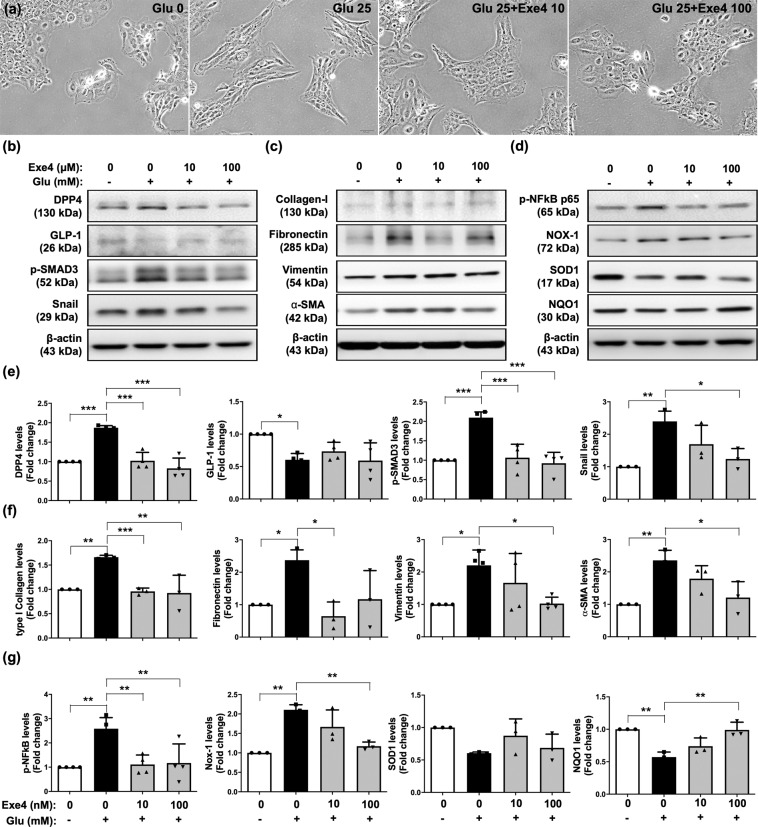
Exendin-4 ameliorated glucose-inducing DPP4 increase, EMT process, inflammation, and oxidative stress in mesothelial cells. **a** Met-5A cells were induced with or without 25 mM D-glucose (Glu) exposure for 120 h. Under 25 mM D-glucose (Glu) induction, Met-5A cells were treated exendin-4 (Exe4) with 0, 10, or 100 nM concentrations, and then observed cell morphology by microcopy at 200×. Scale bars: 50 μm. **b** Western blotting was performed to detect the protein levels of DPP4, GLP-1, p-SMAD3, and Snail. **c** Western blotting was performed to evaluate the protein levels of collagen-I (type I collagen), fibronectin, vimentin, and α-SMA. **d** Western blotting was performed to analyze the protein levels of p-NF-kB, NOX-1, SOD1, and NQO1. **e**–**g** Quantitative results of Fig. 3b, c, and d were shown, respectively. The relative levels were normalized and compared by 0 mM glucose concentration. Data represents the analysis of at least 3 independent experiments and shows mean ± SD. * indicates *p*-value <0.05; ** represents *p*-value <0.01; and *** is *p*-value <0.001.

In the incretin-based therapies, both sitagliptin (DPP4 inhibitor) and exendin-4 (GLP-1 receptor agonist) are widely used to treat DM with glucose-lowering and anti-inflammatory effects^22,33^. Sitagliptin is a tight binding DPP4 inhibitor; exendin-4 is an analog of GLP-1 originally found from the venom of Gila monster with a long half-life, which was also a peptide agonist of the GLP receptor. To evaluate the protective effects of incretin-based therapies against glucose-induced cellular damage, we performed glucose exposure of mesothelial cells with sitagliptin or exendin-4 treatment. Interestingly, both sitagliptin and exendin-4 significantly reversed glucose-induced mesothelial cells into fibroblast-liked morphologies (Figs. 2a, 3a). During EMT process, the changes of cellular morphology correspond with reorganization of filamentous actin (F-actin). Glucose exposure promoted phalloidin-labeled F-actin to form actin stress fibers in the mesothelial cells, but sitagliptin and exendin-4 suppressed the formation of actin stress fibers (Supplementary Fig. 3). Also, sitagliptin and exendin-4 treatments further inhibited the protein expressions of DPP4, soluble DPP4 activity, p-SMAD3, and snail in glucose-exposure situation (Figs. 2b, e, 3b, e, Supplementary Fig. 2a-b).

Furthermore, sitagliptin treatment significantly restored the protein level of GLP-1 during glucose exposure (Fig. 2b), whereas exendin-4 treatment did not exhibit this effect as Sitagliptin (Fig. 3b). On the other hand, the glucose-upregulated protein expressions of mesenchymal markers (type I collagen, fibronectin, vimentin, and α-SMA) were significantly diminished by sitagliptin and exendin-4 treatments (Figs. 2c, f, 3c, f). Consistent with sitagliptin, DPP4 knockdown inhibited the glucose-inducing DPP4 elevation, TGFβ/SMAD3 pathway, and downstream signaling (Supplementary Fig. 4).

On the other hand, sitagliptin and exendin-4 suppressed glucose-induced protein expression of NFκB and NOX-1, and augmented the protein expressions of SOD1 and NQO1 (Figs. 2d,g, 3d, g), indicating sitagliptin and exendin-4 treatments protected mesothelial cells against glucose-induced cellular damage.

### DPP4 deficiency, sitagliptin, and exendin-4 comparably protected peritoneum against CG-induced PF in rats

**Fig. 4:**
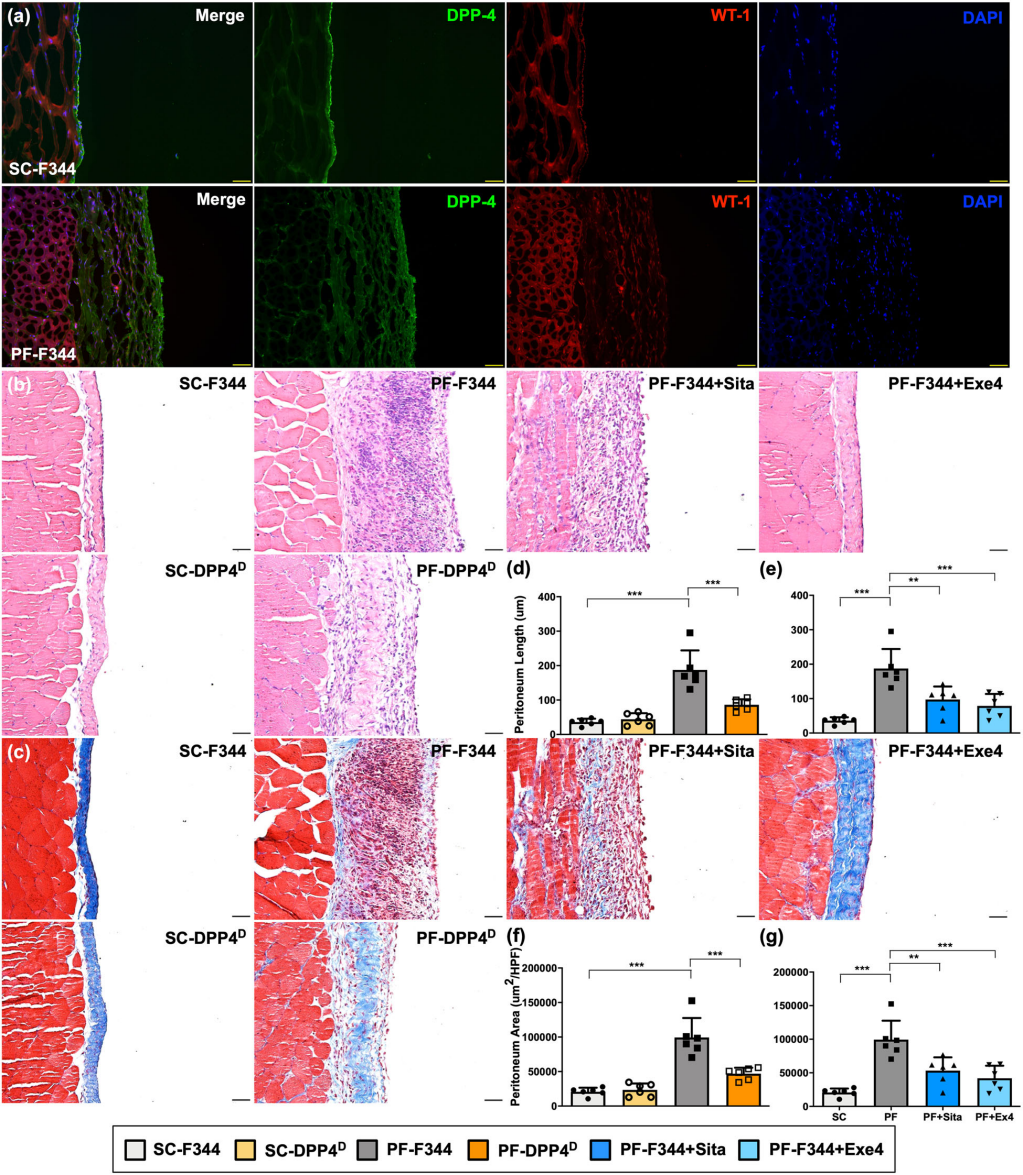
DPP4 deficiency, sitagliptin, and exendin-4 protected peritoneum against CG-inducing peritoneal fibrosis in rats. The experimental model of peritoneal fibrosis (PF) was established by chlorhexidine gluconate (CG) induction for 21 days in wild-type (i.e., Fischer 344) and DPP4 deficient (DPP^D^) rats. **a** Representative images of peritoneal tissue on day 21 were detected by immunofluorescent staining (200×) in SC-F344 (sham control, F344 wild type) and PF-F344 (peritoneal fibrosis, F344 wild type) rats for determining DPP4 (green color) and WT-1 positive signal (red color). DAPI labels cellular nuclei (blue color). **b** Histology of the peritoneum was observed by hematoxylin and eosin staining (H&E staining) in all the groups after CG-inducing PF. **c** Illustrating finding of Masson’s trichrome stain (200×) for identification of peritoneal fibrosis. **d** Quantitative result of peritoneal length by Masson’s trichrome stain in SC-F344, SC-DPP4^D^, PF-F344, and PF-DPP4^D^. **e** Quantitative result of peritoneal length by Masson’s trichrome stain in SC-F344, PF-F344, PF-F344 + Sita (sitagliptin), and PF-F344 + Exe4 (exendin-4). **f**, **g** Quantitative result of peritoneal area by Masson’s trichrome stain in each group. *n* = 6 for each group. Data represents the analysis and shows mean ± SD. Scale bars: 50 μm; HPF: high-power field. ** indicates *p*-value <0.01; *** represents *p*-value <0.001.

To clarify DPP4 role involving in PF in vivo, we next established an experimental model of PF induced by chlorhexidine gluconate (CG) for 21 days in wild-type (i.e., Fischer 344) and DPP4 deficient rats. In sham-control (SC) group, the normal mesothelium was shown as a simple slim layer with positively stained WT-1 attached to connective tissue outside which was obviously detected by immunofluorescent staining (Fig. 4a). On the other hand, submesothelial region thickened and the formation of PF along with DPP4 upregulation in the proliferated peritoneal membrane was clearly identified in CG-treated animals (Fig. 4a, Supplementary Fig. 5).

We further performed H&E staining and Masson trichrome staining to evaluate the morphological feature and severity of fibrosis in the peritoneal membranes, respectively (Fig. 4b, c). Compared to the SC group of F344 animals, the thickness length and area of PF were significantly increased up to 4-fold values in CG-treated F344 rats (*p* < 0.001). In this way, the thickness of PF was substantially reduced (i.e., at least 50%) in DPP4 deficient rats as compared with those of F344 wild-type counterparts in the same condition of CG treatment (Fig. 4c, d, f), suggesting genetic DPP4 deficiency protected peritoneum against CG-induced PF in rodents. In addition, CG-induced peritoneal thickening (i.e., up to 50%) was significantly suppressed in wild-type rats with than without sitagliptin and exendin-4 treatments, once again, indicating an essential role of sitagliptin and exendin-4 treatments on ameliorating the CG-induced PF (Fig. 4b, c, e, g).

### DPP4 deficiency, sitagliptin, and exendin-4 reversed CG-induced EMT process via Smad-3 signaling pathway

**Fig. 5:**
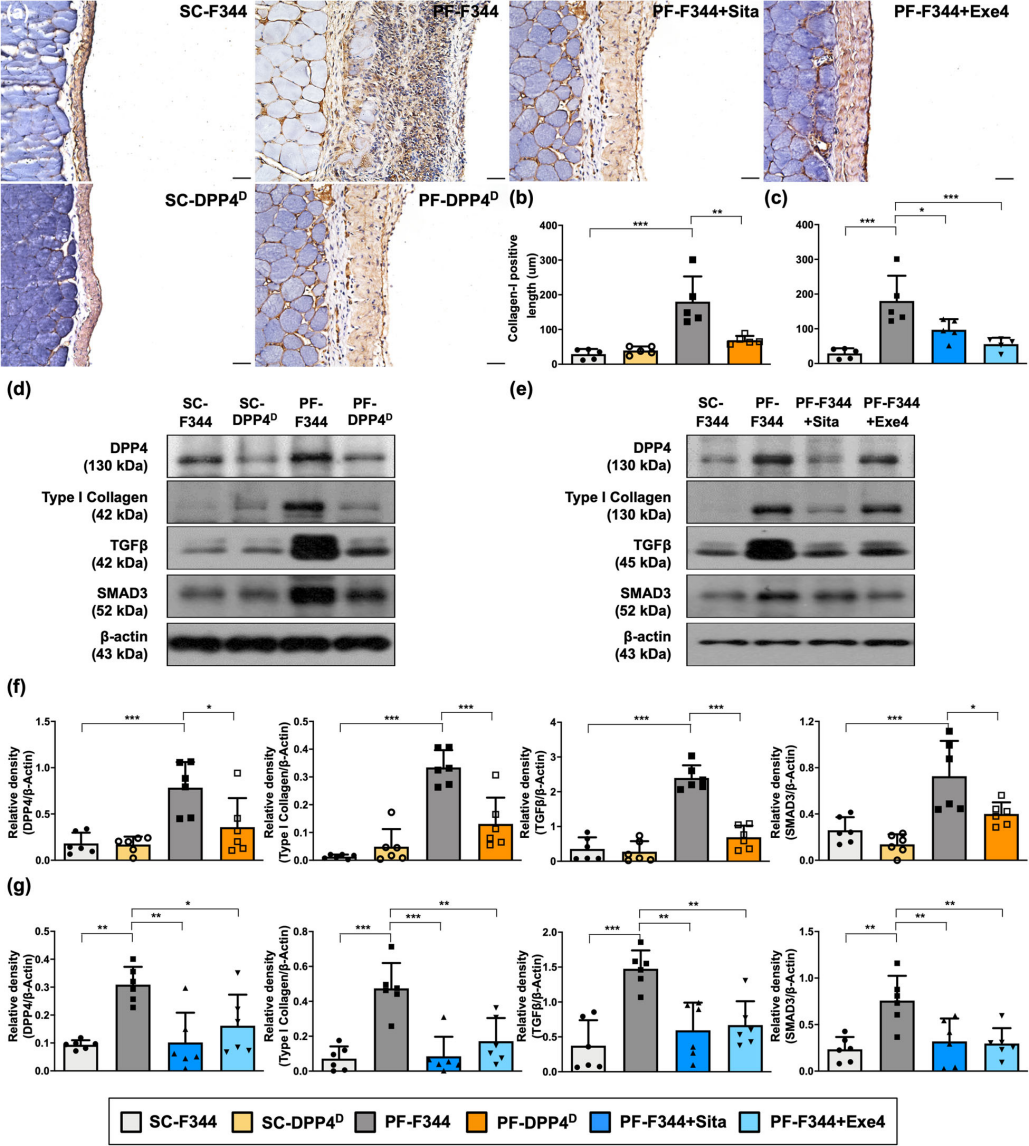
DPP4 deficiency, sitagliptin, and exendin-4 reversed CG-inducing EMT process via SMAD3 pathway in rats. **a** Representative images (200×) of each group for identification of positively stained collagen-I (brown color) by immunohistology staining in peritoneal tissue after CG-inducing PF for day 21. Scale bars: 50 μm. **b**, **c** Quantitative result of positively stained collagen-I length in the peritoneum of each group. **d** Western blotting was performed to detect the protein levels of DPP4, collagen-I, TGF-β, and SMAD3 in the peritoneal tissues of F344 wild type (F344) or DPP4 deficient (DPP4^D^) rats. **e** Western blotting was performed to detect the protein levels of DPP4, collagen-I, TGF-β, and SMAD3 in the peritoneal tissues for evaluating therapeutic protection of sitagliptin and exendin-4. **f**, **g** Quantitative results of Fig. 5d and Fig. 5e were shown, respectively. *n* = 6 for each group. Data represents mean ± SD; * indicates *p*-value <0.05; ** represents *p*-value <0.01; and *** is *p*-value <0.001.

To clarify whether DPP4 participating in EMT process in vivo, we further examined the peritoneal expression of type I collagen, a mesenchymal marker, by immunohistochemical analysis. As expected, microscopic findings demonstrated that the thickness of positively stained type I collagen was significantly increased in PF-F344 group as compared with SC group of wild-type rats, and that was significantly reduced in PF-DPP4 deficient rats (Fig. 5a, b). Meanwhile, the protein level of DPP4 in the peritoneal membrane was significantly enhanced to 4-folds in PF-F344 group as compared with SC-F344 group that was decreased in PF-DPP4 deficient rats again (Fig. 5d).

In situation of peritoneal fibrosis, TGF-β/SMAD3 signaling axis has been recognized to mediate EMT process^34,35^. The results of the present study also displayed the significant upregulations of type I collagen, TGF-β, SMAD3, and Twist in PF-344 group (Fig. 5d, Supplementary Fig. 1). On the other hand, DPP4 deficiency significantly not only suppressed CG-induced protein expression of DPP4 but also downregulated the mesenchymal marker (i.e., type I collagen) and TGF-β/SMAD3 signaling axis (Fig. 5d, f), suggesting DPP4 deficiency alleviated CG-induced PF mainly via reversing EMT process mediated SMAD3 signaling pathway. Similarly, both sitagliptin and exendin-4 significantly inhibited CG-induced the thickness of type I collagen, DPP4 upregulation, and EMT process in peritoneum (Fig. 5a, c, e, g).

### DPP4 deficiency, sitagliptin, and exendin-4 ameliorated CG-induced angiogenesis and inflammation by suppressing NF-κB/MyD88 signaling

**Fig. 6:**
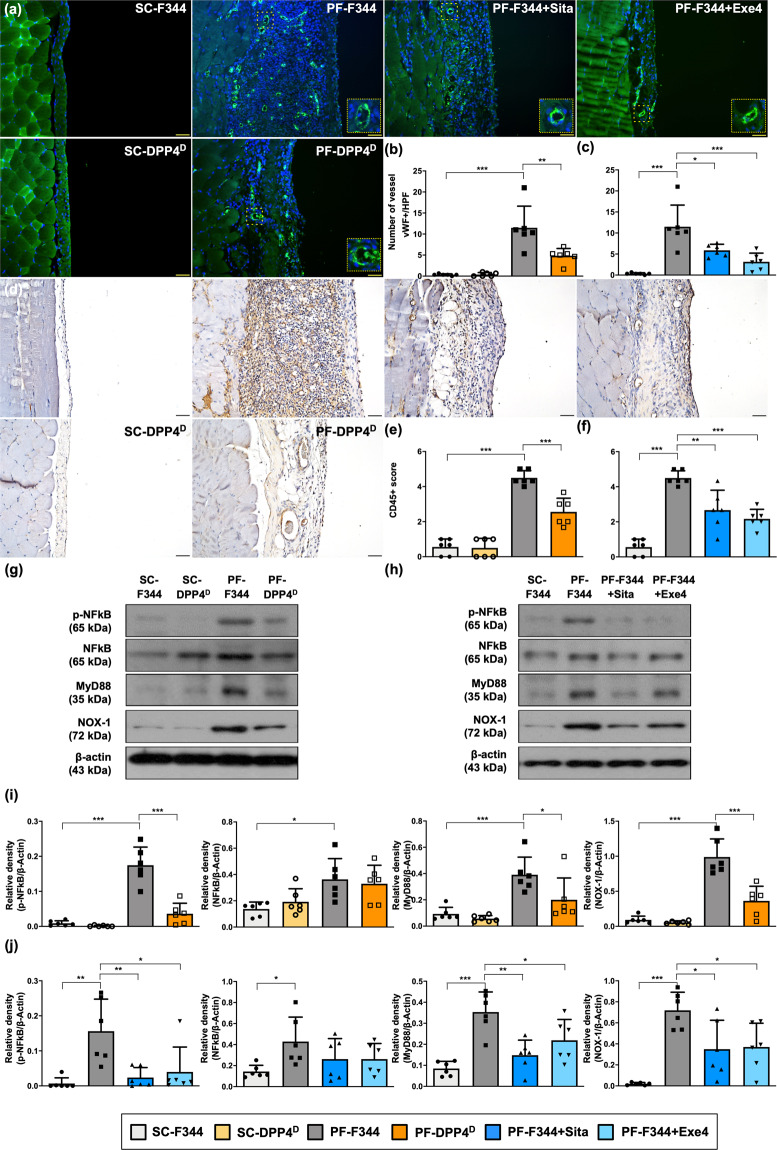
DPP4 deficiency, sitagliptin, and exendin-4 ameliorated CG-inducing angiogenesis and inflammation by suppressing NF-κB/MyD88 signaling in rats. **a** Representative images of vessels in peritoneal tissue on day 21 were detected by immunofluorescent staining (200×) in each group. The blood vessels were indicated by round vWF positive signal (green color). DAPI labels cellular nuclei (blue color). Scale bars: 50 μm. **b**, **c** Quantitative number of vessels as vWF+ per high-power field (HPF) in each group. **d** Illustrating images (200×) of each group for identification of positively stained CD45 (brown color) as the leukocyte marker by immunohistology staining in peritoneal tissue after CG-inducing PF on day 21. Scale bars: 50 μm. **e**, **f** Quantitative of CD45+ score in each group. **g**, **h** Western blotting was performed to detect the protein levels of p-NF-kB, NF-kB, MyD88, and NOX-1 in the peritoneal tissues of each group. **i**, **j** Quantitative results of Fig. 6g and Fig. 6h were shown, respectively. *n* = 6 for each group. Data represents mean ± SD; * indicates *p*-value <0.05; ** represents *p*-value <0.01; and *** is *p*-value <0.001.

Angiogenesis and inflammation, two most common complications in PD patients, contribute to progressively peritoneal remodeling and the event of ultrafiltration failure^36,37^. The immunofluorescent stain exhibited that vessel number was significantly increased in PF-F344 group than in SC-F344 group, and that was reversed in PF-DPP4 deficient group (Fig. 6a, b), suggesting DPP4 deficiency suppressed CG-induced peritoneal angiogenesis. Furthermore, the number of CD45+ cells, an indicator of inflammatory leukocytes infiltrated in peritoneum, was significantly increased in PF-F344 animals than in SC-F344 group, whereas this parameter was reduced in PF-DPP4 deficient group compared with PF-344 group (Fig. 6d, e).

It is well known that the transcription factor NF-κB activation through MyD88 signaling serves as a critical mediator of inflammatory response^38,39^. Consistent with the leukocyte infiltration in CG-induced PF condition, the protein levels of phosphorylated (p)-NF-κB and MyD88 were significantly higher in the peritoneum of PF-344 group than in SC-F344 group. In addition, NOX-1, an indicator of oxidative stress, was also upregulated in the peritoneum of PF-F344 animals (Fig. 6g, i). However, DPP4 deficiency significantly inhibited the levels of p-NF-κB, MyD88, and NOX-1, implying DPP4 deficiency blocked the NF-κB/MyD88 signaling activation, inflammation, and the generation of oxidative stress (Fig. 6g, i).

Moreover, both sitagliptin and exendin-4 treatments significantly diminished CG-induced angiogenesis and leukocytes infiltration in peritoneum (Fig. 6a, c, d, f). On the other hand, sitagliptin and exendin-4 significantly downregulated the NF-κB/MyD88 signaling activation which further reduced the inflammation and oxidative stress (Fig. 6h, j).

### DPP4 deficiency, sitagliptin, and exendin-4 reduced the functional impairments of peritoneal membrane with peritoneal fibrosis in rats

To assess the functional change of the peritoneal membrane, the peritoneal equilibrium test (PET), i.e., a semiquantitative assessment of peritoneal membrane transport function, was performed on day 21. The PET in the present study was based on the previous report^40^ in which the solute transport rates are calculated by the rates of their equilibration between the dialysate and peritoneal capillary blood.

**Fig. 7:**
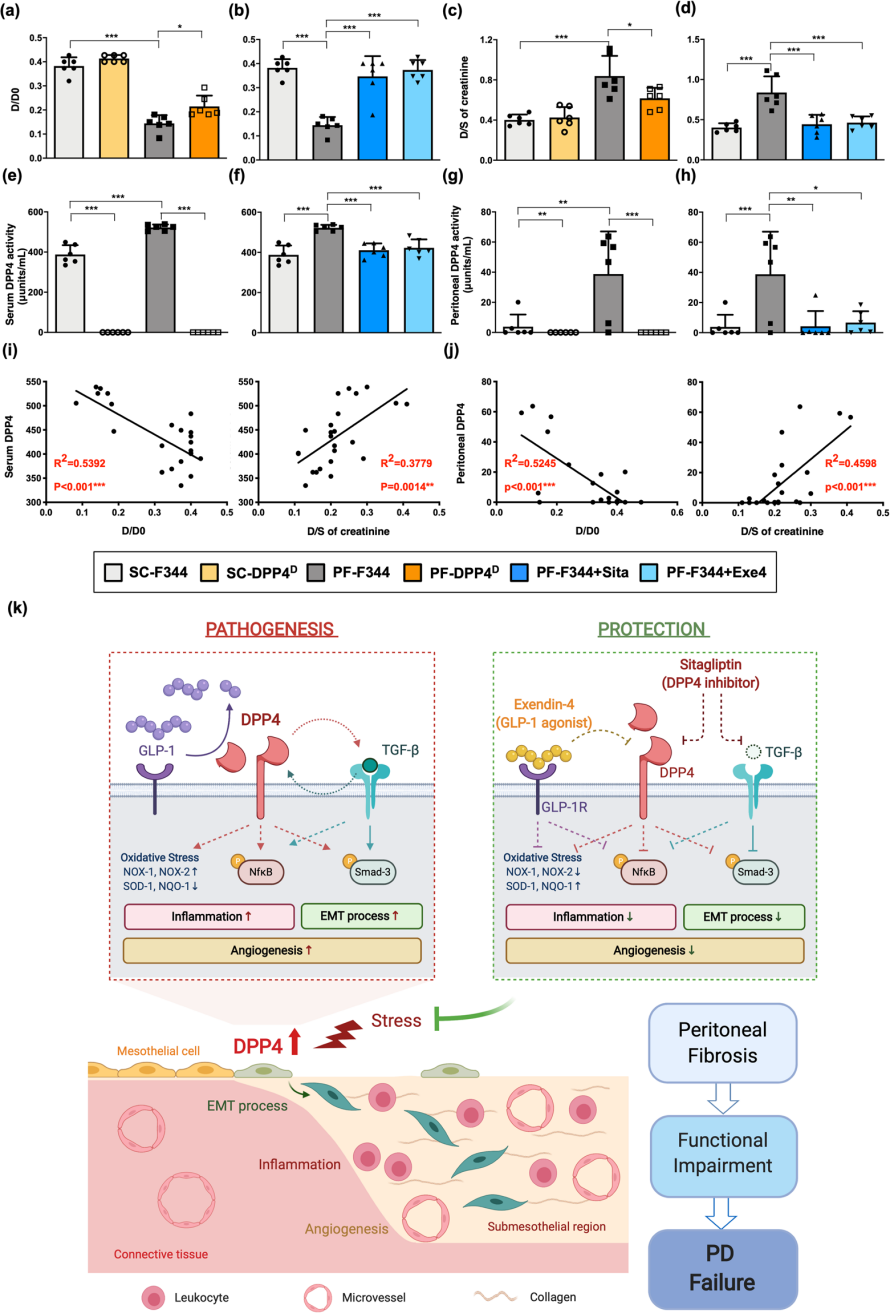
DPP4 deficiency, sitagliptin, and exendin-4 reduced the functional impairments of peritoneal membrane with peritoneal fibrosis in rats. After CG-inducing PF, both F344 wild-type (F344) and DPP4 deficiency (DPP4^D^) rats were injected with dialysate (4.25% Dianeal) at 100 ml/kg body weight to evaluate the peritoneal equilibration test (PET) for assessment of peritoneal membrane transport function. **a**, **b** After injecting dialysate for 1 h, the peritoneal absorption of glucose from the dialysate was monitored the ratio of peritoneal glucose uptake at the end of the test compared to the initiation (D/D0). **c**, **d** Dialysate-to-Serum (D/S) ratio of creatinine was assessed after injecting dialysate for 4 h. **e**, **f** DPP4 activity in serum among groups. **g**, **h** DPP4 activity in peritoneal fluids among groups. **i** In wild-type rats, the correlations between the DPP4 activity in serum and the parameters of peritoneal transport, D/D0 and D/S creatinine, were evaluated. **j** In wild-type rats, the correlations between the DPP4 activity in peritoneal fluids and the parameters of peritoneal transport were calculated. *n* = 6 for each group. Data represents mean ± SD; * indicates *p*-value <0.05; ** represents *p*-value <0.01; and *** is *p*-value <0.001. **k** The schematic illustrating the DPP4 role involving in the pathogenetic progression of peritoneum to PD failure, which was established from our experimental results and clinical observation. During glucose exposure or CG-inducing injury, DPP4 was upregulated and degraded active GLP-1. Meanwhile, DPP4 exhibited the crosstalk with TGF-β to further trigger EMT process via SMAD3 pathway. On the other hand, DPP4 upregulation induced the oxidative stress generation and activation of NFκB, pathway followed by attract inflammatory cell infiltration. Both inflammation and EMT progressed angiogenesis as well as caused collagen deposition, peritoneal fibrosis, functional impairment, and finally PD failure. Importantly, the incretin-based therapy, including sitagliptin and exendin-4, effectively protected peritoneal integrities and improved the peritoneal impairments. This schematic was created with BioRender.com. EMT epithelial–mesenchymal transition, PD peritoneal dialysis.

The results of our study demonstrated that the absorption rate of glucose from the dialysate (D/D0) and the transport rate of blood creatine from the serum (D/S) were significantly higher in CG-treated rats (i.e., PF-F344) than in the F344 rats treated with the saline alone (Fig. 7a, c). The peritoneal membrane in PF-F344 group possessed an increased permeability that caused a significantly rapid loss of the osmotic gradient between dialysate and capillary. This finding implicated that the CG exposure damaged not only the peritoneal delicate ultrastructure but also its functional integrity in PF-F344 animals, as well as remarkably increased DPP4 activities in serum and peritoneal fluids (Fig. 7e, g). On the other hand, the DPP4 deficiency acted as an important contributor for improving the peritoneal membrane function in CG-treated DPP4 deficient rats (Fig. 7a, c, e, g).

As the condition of DPP4 deficiency, sitagliptin or exendin-4 treatment significantly attenuated DPP4 activity, peritoneal injury, and functional impairment in PF-F344 animals (Fig. 7b, d, f, h).

We further analyzed the correlation between DPP4 activity and peritoneal function in wild-type rats (i.e., F344). The result revealed that DPP4 activity in serum was significantly positively correlated with the glucose absorption (D/D0) and the transport rate of creatine, indicating DPP4 activity in circulation was closely linked to the functional impairments of the peritoneal membrane (Fig. 7i). Finally, we found that the DPP4 activity in peritoneal fluid had more significant correlation with the peritoneal membrane dysfunction (Fig. 7j). Taken together, both sitagliptin and exendin-4 treatments could significantly attenuate peritoneal injury and dysfunction that were closely related to DPP4 activation.

## Discussion

So far, the mechanism of peritoneal injury caused by long-term PD and the corresponding potential therapeutic modality are not yet clearly established. One of the essential findings in the present study was that we identified the DPP4 played a key role in peritoneal fibrosis and dysfunction through promoting EMT, inflammation, and angiogenesis. Furthermore, sitagliptin and exendin-4 treatments markedly inhibited DPP4 levels, resulting in ameliorating peritoneal fibrosis and functional impairment. In particular, finding from nationwide registered data showed DPP4 inhibitors significantly prevent PD failure in ESRD patients, highlighting the great therapeutic benefits of DPP4 inhibitors to PD patients.

The fundamental finding of PD solution-induced peritoneal injury and fibrosis is that the distinctively histopathological change of the mesothelial cells undergoes EMT process^31,32^. In EMT process, mesothelial cells undergo trans-differentiation and convert the epithelial to mesenchymal phenotype, which is characterized as loss of cellular adherent and junction, acquisition of fibroblastic morphology and ability, proliferation of α-smooth muscle actin (α-SMA)-positive myofibroblasts, accumulation of collagen and increase of the thickness in submesothelial compact zone^15,41^. In addition, TGF-β delivers pro-EMT signals and further induces Smad2/3 activation, followed by stimulating snail to transcript mesenchymal molecules and promoting the formation of EMT^42,43^. An essential finding in the present study was that the high concentration of glucose triggered EMT process with fibrotic phenotype via activating p-SMAD3 which in turn further stimulated snail, followed by increasing the mesenchymal markers (i.e., collagen-I, fibronectin, α-SMA, vimentin) and decreasing the epithelial marker (i.e., ZO-1) in mesothelial cells. Moreover, DPP4 deficiency or DPP4 inhibitors repressed TGF-β levels (Fig. 3d–g). Interestingly, TGF-β also triggered an increase of DPP4 levels followed by regulating downstream signaling (Supplementary Fig. 6), suggesting that DPP4 and TGFβ exhibited the crosstalk regulation. In this way, our findings were consistent with the findings of previous studies^15,41–43^.

Another essential finding in the present study was that high concertation of glucose remarkably upregulated DPP4 level in mesothelial cells, resulting in initiating the propagation of the EMT process, inflammation, and generation of oxidative stress. Of important finding was that these molecular-cellular perturbations were markedly suppressed by incretin-based therapies (i.e., sitagliptin and extendin-4). Accordingly, our findings strengthened the findings of previous studies^15,41–43^.

Previous study has demonstrated that an increase in the number of blood vessels in the peritoneal tissues of PD patients was commonly observed and more pronounced in those of PD patients with peritoneal membrane ultrafiltration failure^44^. Consistent with the previous study^44^, our study demonstrated a progressive increase in submesothelial thickening (i.e., through EMT process), in number of blood vessels and in a unique vasculopathy after CG-treated F344 PF.

On the other hand, DPP4 possesses soluble and membrane-bound DPP4 form, which share the main domains including the catalytic site and protease to degrade a broad range of substrates with many biological actions. Except to DPP4 enzymatic pathway, membrane-bound DPP4 possesses the cytoplasmic, transmembrane and flexible domains, that can interact with extracellular matrix components and further regulate intracellular signal transduction. In normoglycemic conditions, Panchapakesan and Pollock (2014) reveal the membrane-bound DPP4 rarely or does not interact with the cation-independent mannose 6-phosphate receptor (CIM6PR), but this interaction increases in hyperglycemic conditions causing to the activation of TGF-β pathway and renal fibrosis^45^. Moreover, Zeisberg and Zeisberg demonstrate the interaction between DPP4 and integrin β1 would initially elicit the endothelial cells into endothelial–mesenchymal transition and finally into fibrosis through activating Smad-3 pathway in kidney injury^46^. This nonenzymatic regulation of DPP4 influences the cellular signal transduction, which could be explained how to influence TGF-β/Smad-3 pathway in this study^47^. Since DPP4 exhibits several pleiotropic effects not only by enzymatic but also by nonenzymatic pathways, DPP4 has been found to play an essential role in a variety of disease entities and states, such as inflammation, cancer, diabetes, hepatic, and renal fibrosis^48–50^. In type 2 diabetes, plasma DPP4 activity is increased for glycemic deregulation^51^. In addition, DPP4 activities in serum level and peritoneal fluids were revealed to be remarkably increased in CG-treated F344 PF. In this way, our finding corroborated with the finding of previous study^51^.

A principal finding in the present study was that in CG-treated DPP4 deficiency rats, the DPP4 activity was extremely diminished, and that in turn substantially attenuated EMT, angiogenesis, inflammatory reaction, and generation of oxidative stress, resulting in protecting the peritoneal structure and function. Another principal finding in the present study was that a strong correlation between DPP4 activities in serum or in peritoneal fluids and functional impairment of peritoneal membrane, implying DPP4 activity would be a unique biomarker for predicting the PD outcome. The crucial finding in the present study was that the incretin-based therapies (i.e., sitagliptin and extendin-4) offered an identical effect as DPP4 deficiency in CG-treated F344 animals on ameliorating the inflammation, angiogenesis, EMT process, and generation of oxidative stress, and protecting the peritoneum against CG-treated injury. The most important finding in the present study was that our nationwide registered data of diabetic ESRD patients showed the incidence of PD failure was significantly lower in with than without receiving DPP4 inhibitors. Since DPP4 inhibitors have been recommended in treating hyperglycemia in diabetes patients on PD for several favorable characteristics^52^, it is important to clarify whether DPP4 was involved in peritoneal fibrosis. Among all collected types of antidiabetic drugs, diabetic ESRD patients with DPP4 inhibitors treatment had the lowest incidence of PD failure, highlighting that the utilization of DPP4 inhibitors should be strongly considered as a priority of our daily clinical practice for protecting the PD and extending its lifespan. We believe our study provided solid data to establish the role of DPP4 in the pathogenesis of peritoneal fibrosis and explain the beneficial effects of DPP4 inhibitors fundamentally.

GLP-1 is an incretin and possesses hypoglycemic ability in a glucose-dependent manner by enhancing insulin^53^. In circulation, endogenous GLP-1 is rapidly cleaved by soluble DPP4, which is suggesting that the regulation between DPP4 and GLP-1 may involve in the important physiological or pathological roles^21^. Based on this concept, two kinds of incretin-based therapies, including DPP4 inhibitors and GLP-1 agonist, have been developed for the treatment of type 2 diabetes. In this study, we found the increase of DPP4 accompanied with the decrease of active GLP-1, but the decrease of active GLP-1 levels did not reach statistical significance during CG-inducing fibrosis in rats (Supplementary Fig. 7a-b). In addition, peritoneal impairments were significantly associated with DPP4 activities (Fig. 7) but not with active GLP-1 levels (Supplementary Fig. 7c), suggesting DPP4 plays a critical role than GLP-1 in the status of peritoneal injury.

In conclusion, our results of cell level and animal model studies, and clinical observational findings showed that DPP4 enzyme played a key contributor to the peritoneal fibrosis and functional impairment, whereas DPP4 inhibitors safeguarded PD from dysfunction and failure.

## Methods

### Study population and primary outcome of PD transition to HD

The clinical part in the present study was analyzed from Taiwan National Health Insurance Research Database, which contained complete healthcare information of 23.74 million Taiwanese collected by Taiwan National Health Insurance program and has been considered as a reliable database for population analysis^54^.

We conducted the retrospective population-based cohort study (1997–2013). A total of 174,686 ESRD patients with type 2 diabetes and age between 18 and 80 years were identified from one million general populations after excluding those who have history of malignancy and liver cirrhosis, age of younger than 18 years, and follow-up period <1 year. The ESRD patients were further divided into three groups, including 19,828 patients with PD, 171,266 with HD, and 6787 with RT. We further defined the study and comparison groups according to user and nonuser of DPP4 inhibitors. Comparison group was selected in a 1 to 10 ratio by matching study group with age, gender, socioeconomic status, and relevant medications. The users of DPP4 inhibitor were defined as diabetic patients with DPP4 inhibitor prescribed for sugar control for more than three months (*n* = 16,048). On the contrary, those diabetic subjects with oral antidiabetic agents other than DPP4 inhibitor or use of DPP4 inhibitor for less than 3 months were defined as the nonusers of DPP4 inhibitor (*n* = 158,638).

We compared the incidence of PD transition to HD between users and nonusers of DPP4 inhibitors. Those ESRD patients who changed dialysis modality from PD to HD for any reason, e.g., PD catheter infection or fibrosis and peritonitis, were identified as PD failure with a need for PD transition to HD. The diagnoses were verified with ICD-9-CM codes and procedure charge/codes. The study design was approved by the Ethics Institutional Review Board of Chang Gung Memorial Hospital (No. 201702246B1)

### The distribution of antidiabetic drugs administered for patients with ESRD on different renal replacement therapies

In order to further study the rate of PD transition to HD among different type of antidiabetic drugs, detailed information of prescription was retrieved based on Anatomical Therapeutic Chemical classification system (WHO ATC codes). Information regarding glucagon-like peptide-1 (GLP-1) agonists was not listed because of long-term follow-up data is unavailable in Taiwan, i.e., these agents only appeared on the Taiwan market after 2013. Distribution of antidiabetic drugs prescribed for the diabetic patients with ESRD on PD was investigated initially, and then the rate of PD transition to HD in subgroups of different antidiabetic drugs was followed up till the end of 2013.

### Mesothelial cell culture

Mesothelial cells, Met-5A cells (65302), were purchased from Bioresource Collection and Research Center (BCRC) in Taiwan. The BCRC cell bank is a nonprofit organization supported by the Taiwan government and has a strict quality control system, including sterility, mycoplasma contamination tests, and STR profiling analysis for each banked cell line. Met-5A cells were maintained in the M199 culture medium supplemented with penicillin (100 U/ml), streptomycin (100 μg/ml), and 10% fetal bovine serum (FBS) in 37 °C incubator at a humidified atmosphere of 95% air and 5% CO_2_. To determine the impact of high-glucose concentration (G8270, Sigma) on the molecular-cellular signaling, Met-5A cells were incubated with serum-free medium for 24 h for arrest and synchronization of the cell growth. After this time period, Met-5A cells were treated with serum-free medium containing various concentrations of glucose for 120 h. To evaluate therapeutic benefit, Met-5A cells were treated with or without sitagliptin (PHR1857, Sigma) or exendin-4 (Byetta, AstraZeneca) under glucose induction. In addition, TGF-β1 treatment was utilized as a positive control of EMT, 2 ng/ml TGF-β1 recombinant protein (240-B, R&D Systems) for 120 h, and cell morphology observed by microcopy at 200×.

### Western blotting

These experimental protocols were based on our previous study^55^. The 50 μg protein extracts were loaded and separated by SDS-PAGE. The SDS-PAGE gel was next transferred to a polyvinylidene difluoride membrane (GE, UK), followed by blocking overnight. The membranes were incubated with optimal primary antibodies and horseradish peroxidase-conjugated secondary antibodies. Immunoreactive bands were identified by enhanced chemiluminescence (WBKLS0500, Millipore) and exposed to Biomax L film (Kodak) or detected by UVP BioSpectrum Imaging System (BioSpectrum 810). The semiquantitative signals were finally analyzed by Labwork software (UVP, Waltham) or ImageJ. All the antibody information and conditions were listed in Supplementary Table 1.

### Animal model and animal grouping

This animal procedure was approved by the Institute of Animal Care and Use Committee at Kaohsiung Chang Gung Memorial Hospital (Affidavit of Approval of Animal Use Protocol No. 2017111301) and performed according to the guidelines for the care and use of laboratory animals. The pathogen-free, adult male, 8–10 weeks of age, wild-type Fischer 344 (F344) rats, and DPP4 deficient rats (DPP4^D^, i.e., F344/DuCrlCrlj strain rats with a missense mutation (Gly633Arg) in DPP4 gene leading to DPP4 deficiency and lack DPP4 activity) were provided from Charles River Technology (BioLASCO Taiwan Co., Ltd) and utilized as study animals.

These animals were housed with 23–24 °C controlled temperature and 12/12 h light/dark cycles in animal center of our hospital, which was an animal facility approved by Association for Assessment and Accreditation of Laboratory Animal Care International (AAALAC; Frederick, MD, USA).

The PF was established by daily intraperitoneal injection of 0.1% chlorhexidine gluconate (CG) in saline at a dose of 10 ml/kg body weight into the peritoneal cavity for 21 consecutive days. This experimental model of PF was based on the previous study^56^ with some modification. Sham-control group of animals were injected with an equal amount of 0.9% saline. On day 2 after CG-induced PF, 600 mg/kg sitagliptin (i.e., a DPP4 inhibitor, Januvia®, MSD) was orally administrated into each wild-type F344 rat daily for 20 days. On the other hand, the 10 μg/kg exedin-4 (GLP-1 receptor agonist, Byetta, AstraZeneca) was intra-peritoneally injected for rat daily for 20 days after CG injection for 1 day. At day 21 after CG-induced PF, all rats were euthanatized by CO_2_ inhalation, and the parietal peritoneum were harvested away from the injection points for further analysis.

Animals were categorized into six groups (*n* = 6 each group): (1) sham control F344 (SC-F344); (2) SC of DPP4 deficiency rats (SC-DPP4^D^); (3) PF-induced F344 rats (PF-F344); (4) PF-induced DPP4 deficiency rats (PF-DPP4^D^); (5) PF-induced F344 rats with daily 600 mg/kg sitagliptin (PF-F344 + Sita) for 20 days; and (6) PF-induced F344 rats with intraperitoneal injection of 10 μg/kg exedin-4 (PF-F344 + Exe4)/day for 20 consecutive days.

### Histological study of fibrosis area and immunofluorescent staining

These experimental protocols were based on our previous report^57^. Rehydrated paraffin sections were treated with 3% H_2_O_2_ for 30 min and then incubated with blocking by 5% BSA in PBS for 30 min at room temperature, followed by incubation with primary and secondary antibodies. Masson’s trichrome staining (TRM-2, ScyTek) was used to study fibrosis in peritoneal membrane specimens. The 4-μm-thick serial sections of specimens were performed by Cryostat (Leica CM3050S). The integrated area (μm^2^) of peritoneal fibrosis in each section was analyzed by Image Tool 3 software (University of Texas). In each section, three randomly selected HPFs (100×) were detected and averaged for each animal to determine the fibrotic length or area (cellSens Standard, Olympus). All the antibody information and conditions were listed in supplementary Table 1.

### Assessment of DPP4 enzyme activity

DPP4 activity (MAK088, Sigma) in circulation and in peritoneal fluids was assessed and calculated by using standard methods according to manufacturers’ instructions.

### Peritoneal equilibrium test

A peritoneal equilibrium test was conducted based on previous study and performed on day 21^58^. Prior to animals to be euthanized, the rats in each group were instilled with PD solution (DIANEAL PD-2 Peritoneal Dialysis Solution 4.25% Dextrose, 5B9896, Baxter) at 100 ml/kg body weight. At the end of time point, we collected the dialysate samples through midline incision via insertion of a dialysis catheter. The peritoneal permeability of glucose D/D0 [the ratio of time after initiation (D) to 0-h (D0) glucose concentration of the dialysate] was calculated and determined as the peritoneal absorption of glucose from the dialysate. The creatinine ratio was calculated as dialysate (D)-to-serum (S) ratio (D/S urea) of blood urea nitrogen.

### Statistical analysis and reproducibility

Quantitative data were expressed as mean ± SD. To analyze the differences of mean among groups, ANOVA analysis was adequately performed, followed by Turkey’s multiple comparison test. In addition, student *t*-test was assessed between two groups. Transition rate of PD to HD was evaluated with Chi-Square test. Correlation estimate was analyzed by Pearson correlation coefficients. PASW Statistics 18 SPSS18 (IBM) software and GraphPad Prism (GraphPad Software v.8.0) were utilized. A probability value <0.05 with two-tailed statistical test was considered as statistically significant. Sample sizes and number of replicates were described in figure legends.

### Reporting summary

Further information on research design is available in the Nature Research Reporting Summary linked to this article.

## Supplementary information

Supplementary Information

Description of additional supplementary files

Supplementary Data 1

Reporting Summary

## Data Availability

All relevant data underlying the graphs and charts were presented in Supplementary Data 1. The uncropped blots were shown in figures are provided in Supplementary Fig. 8.
