# Supplementary material for: Dipeptidyl peptidase 4 promotes peritoneal fibrosis and its inhibitions prevent failure of peritoneal dialysis
Source: Commun Biol. 2021 Jan 29;4:144. doi: 10.1038/s42003-021-01652-x (PMC7846859; doi:10.1038/s42003-021-01652-x)
Supplement: Supplementary file 1 — Supplementary Information [file 42003_2021_1652_MOESM1_ESM.pdf]

Supplementary Figure

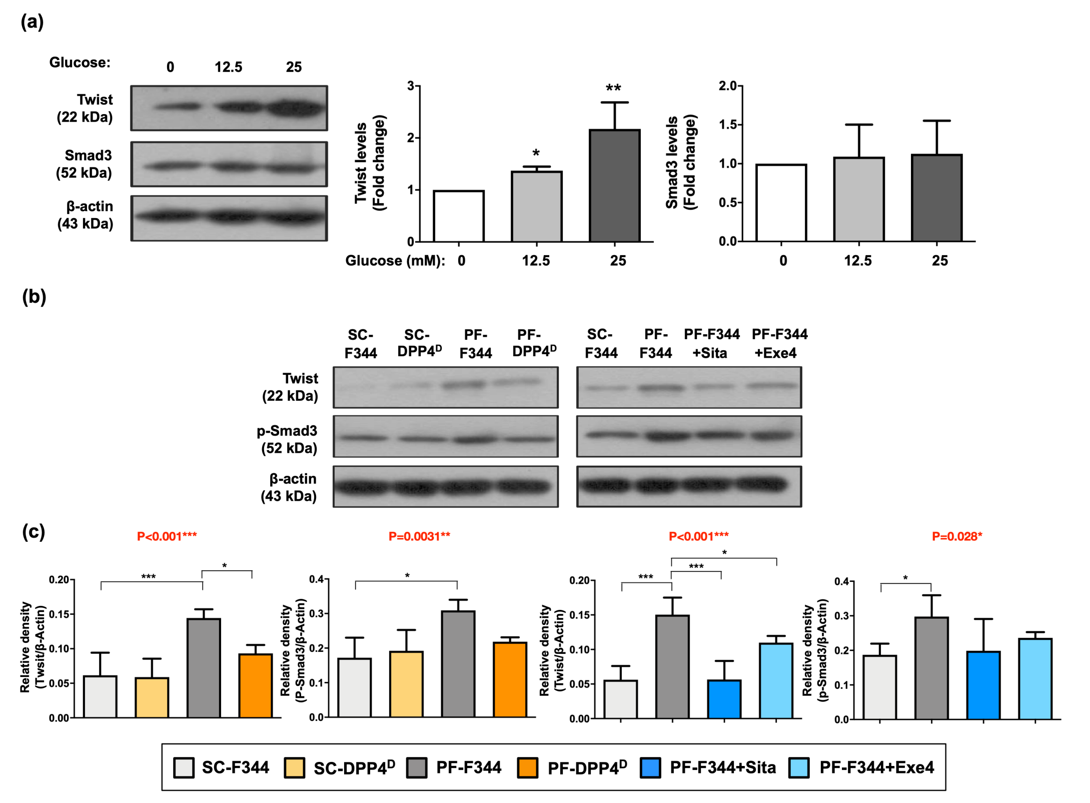

Supplementary Figure 1. The twist, Smad3 or p-Smad3 protein levels were shown under glucose treatment or in peritoneum against CG-inducing peritoneal fibrosis in rats. (a) Met-5A cells were exposed in 0, 12.5, and 25 mM D-glucose for 120 hr as well as performed to detect the protein levels of twist and Smad3 by Western blotting. The quantitative results were shown in the right panel. The relative levels were normalized and compared by 0 mM glucose concentration. Data represents the analysis of 3 independent experiments. (b) The experimental model of peritoneal fibrosis (PF) was established by chlorhexidine gluconate (CG) induction for 21 days in wild type (i.e., Fischer 344) and DPP4 deficient (DPP^{D}) rats. Western blotting was performed to detect the protein levels of twist and p-Smad3 in the peritoneal tissues of each group. (c) Quantitative results of Supplementary Fig.1b was shown respectively. n = 6 for each group. Data represents mean ± SD; * indicates p-value< 0.05; ** represents p-value< 0.01; and *** is p-value< 0.001.

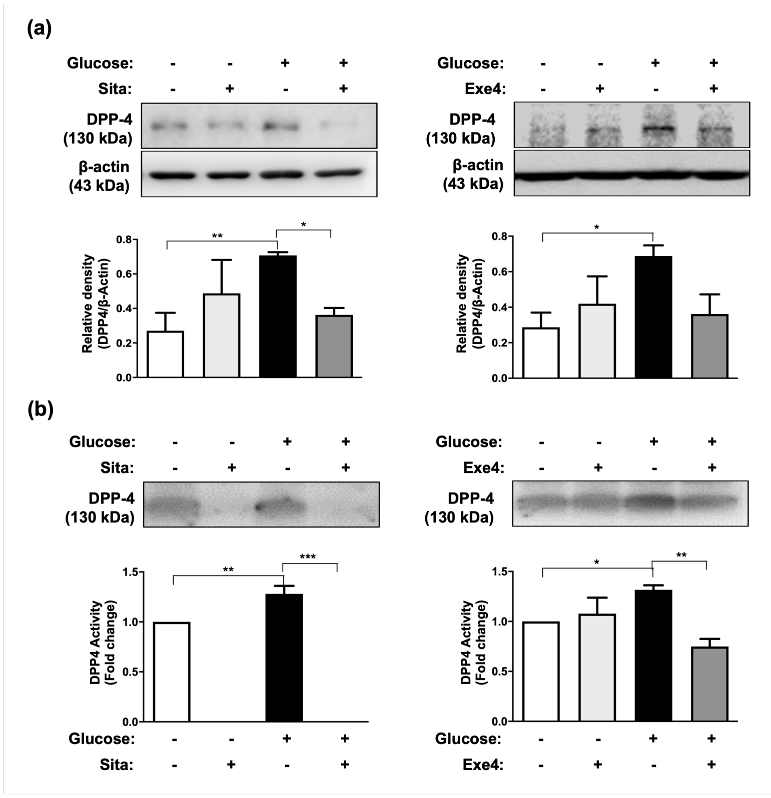

Supplementary Figure 2. Sitagliptin and exendin-4 repressed the increases of DPP4 protein levels and activities, which were triggered by glucose exposure. (a) Met-5A cells were incubated with and without 25 mM D-glucose exposure for 120 hr. Under 25 mM D-glucose exposure, Met-5A cells were treated with and without 100 μM sitaglipin (Sita) or 100 nM exendin-4. Western blotting was performed to detect the protein levels of cellular DPP4. The quantitative results were shown in the above panel. (b) To examine extracellular DPP4, we collected the supernatant of Met-5A for 5 gays glucose exposure. The upper panels indicated the DPP4 protein levels evaluated by Western blot in the supernatant. The lower panel represented DPP4 activities in the supernatant. The relative levels were normalized by 0 mM glucose concentration without drug treatments. Data represents the analysis of 3 independent experiments. Data represents mean ± SD; * indicates p-value< 0.05; ** represents p-value< 0.01; and *** is p-value< 0.001.

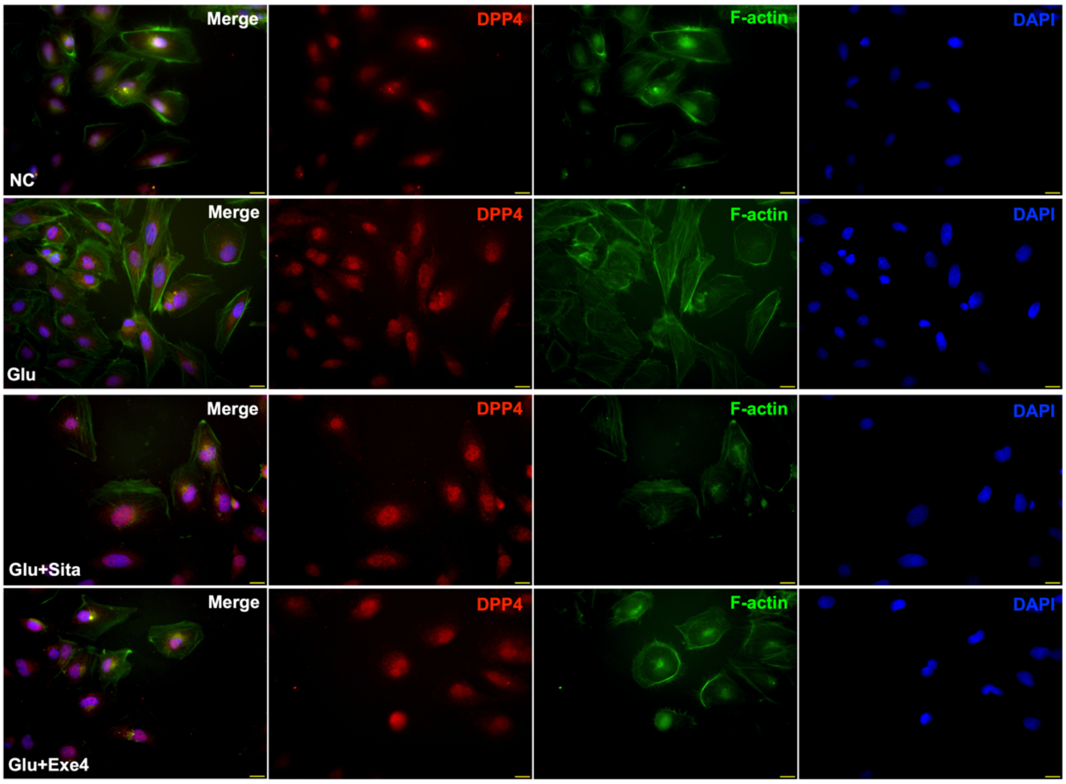

Supplementary Figure 3. Sitagliptin and exendin-4 suppressed the formation of actin stress fibers triggered by glucose exposure. Met-5A cells were incubated with and without 25 mM D-glucose exposure (Glu) for 120 hr. Under 25 mM D-glucose exposure, Met-5A cells were treated with 100 μM sitaglipin (Sita) or 100 nM exendin-4 (Exe4). Representative images of immunofluorescence staining were detected in different groups for determining phalloidin-labeled F-actin (green color) and DPP4 signal (red color). DAPI labels cellular nuclei (blue color). Scale bars: 20 μm. The represented images showed the one of results of 3 independent experiments.

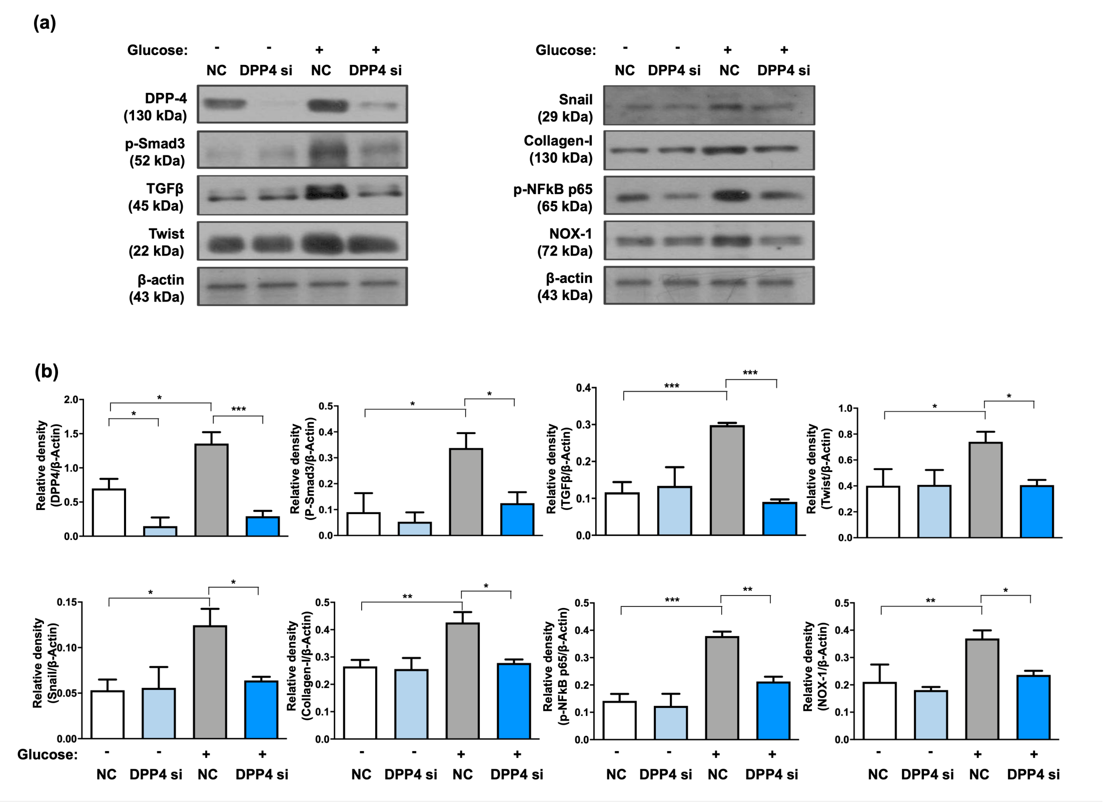

Supplementary Figure 4. DPP4 siRNA knockdown suppressed glucose-inducing DPP4 expression, EMT process, inflammation and oxidative stress in mesothelial cells. (a) In the situation with DPP4 siRNA knockdown (DPP4 si) and without knockdown (negative control, NC), Met-5A cells were incubated with and without 25 mM D-glucose exposure for 120 hr to survey the protein expressions. Western blotting was performed to detect the protein levels of DPP4, p-Smad3, TGF-β, twist, snail, collagen-1, p-NF-kB, and NOX-1. (c) Quantitative results of Supplementary Fig.4a was shown respectively. Data represents the analysis of 3 independent experiments. Data represents mean ± SD; * indicates p-value< 0.05; ** represents p-value< 0.01; and *** is p-value< 0.001.

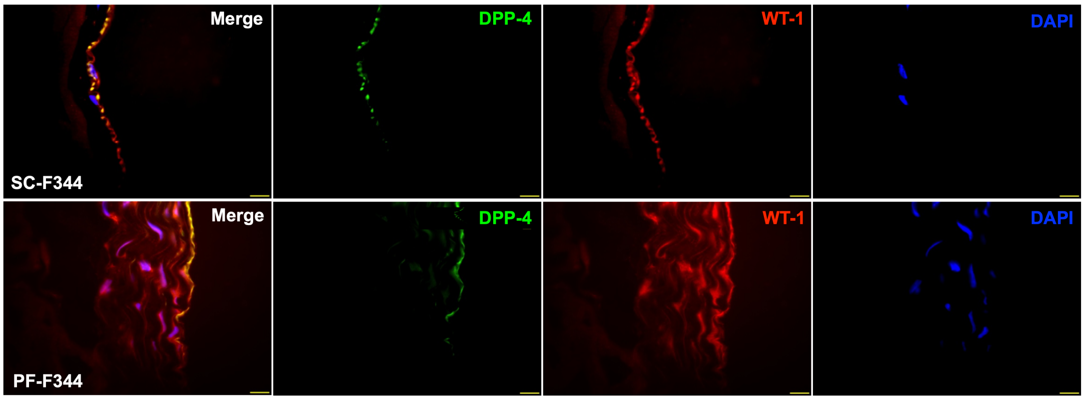

Supplementary Figure 5. Peritoneal sub-mesothelial region thickened accompanying with DPP4 upregulation in CG-treated animals. The experimental model of peritoneal fibrosis (PF) was established by chlorhexidine gluconate induction for 21 days in wild type (i.e., Fischer 344) and DPP4 deficient (DPP^{D}) rats. Representative images of peritoneal tissue on day 21 were detected by immunofluorescent staining (1000x) in SC-F344 (sham control, F344 wild type) and PF-F344 (peritoneal fibrosis, F344 wild type) rats for determining DPP4 (green color) and WT-1 positive signal (red color). DAPI labels cellular nuclei (blue color). Scale bars: 10 μm. The represented images showed the one of results of 3 individual tissues.

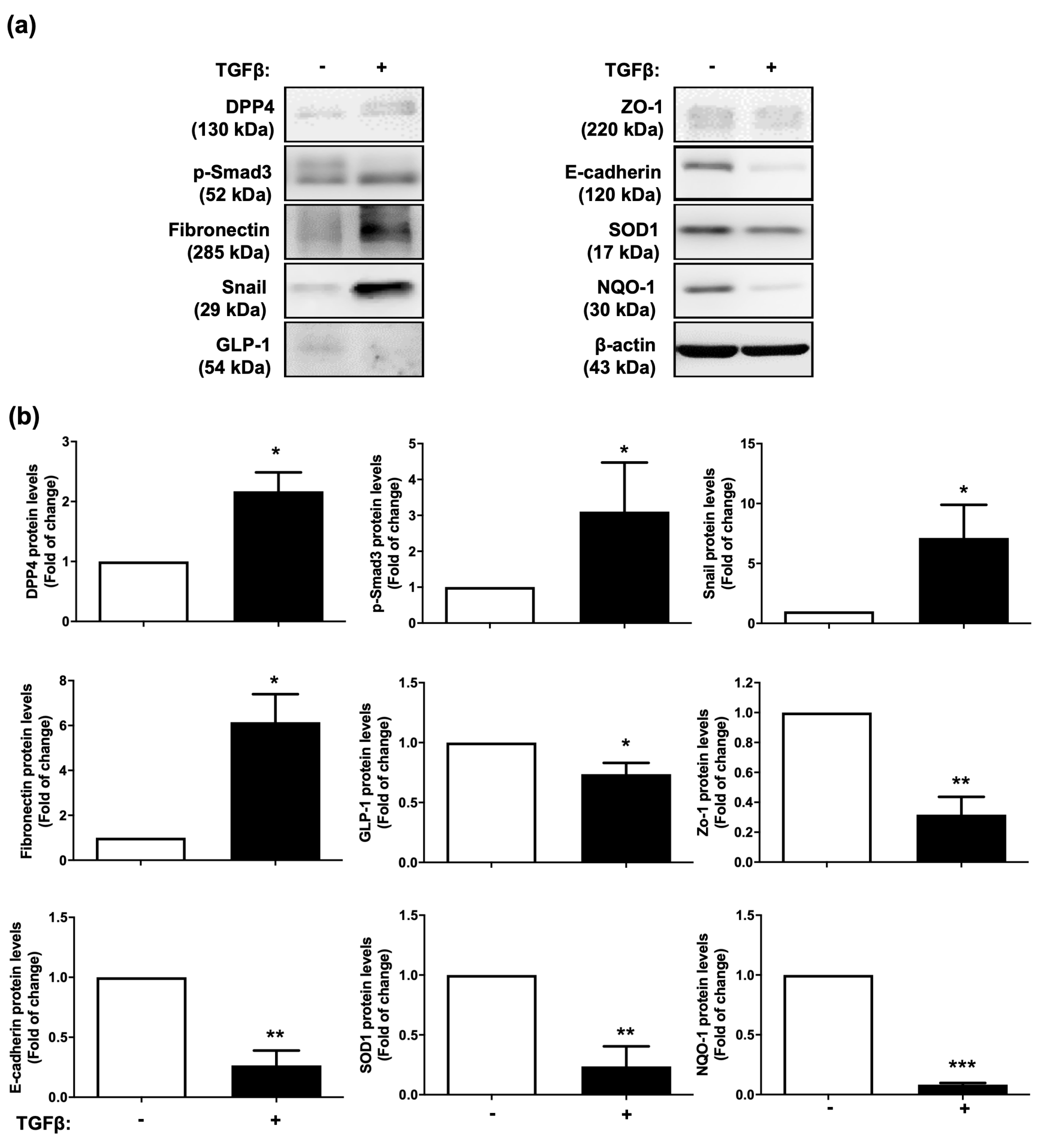

Supplementary Figure 6. TGF-β triggered the increase of DPP4 levels followed by regulating downstream signaling. Met-5A cells were incubated with and without 2 ng/mL TGFβ treatment for 120 hr. (a) Western blotting was performed to detect the protein levels of DPP4, p-Smad3, Fibronectin, snail, GLP-1, ZO-1, E-cadherin, SOD-1 and NQO-1 in Met-5A cells. (b) Quantitative results of Supplementary Fig.6a was shown respectively. Data represents the analysis of at least 3 independent experiments. Data represents mean ± SD; * indicates p-value< 0.05; ** represents p-value< 0.01; and *** is p-value< 0.001.

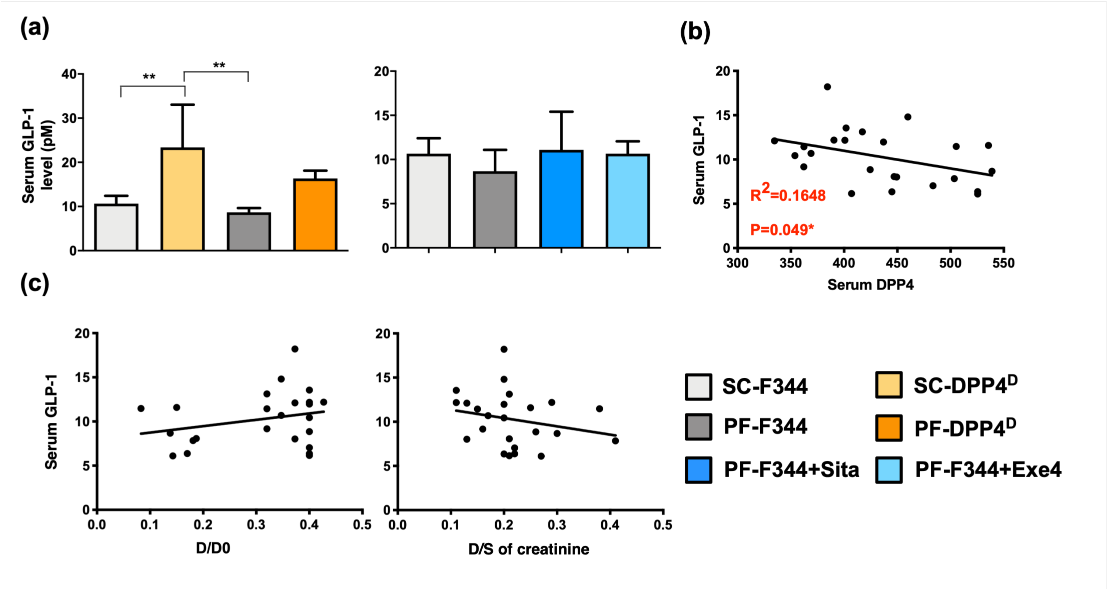

Supplementary Figure 7. Active GLP-1 levels and the correlations in the circulation of rats. The experimental model of peritoneal fibrosis (PF) was established by chlorhexidine gluconate (CG) induction for 21 days in wild type (i.e., Fischer 344) and DPP4 deficient (DPP^{D}) rats. (a) The left panel showed the quantitative result of active GLP-1 levels in serum of SC-F344, SC-$DPP4^{D}$, PF-F344 and PF-$DPP4^{D}$groups. Additionally, the right panel indicated the quantitative result of active GLP-1 levels in serum of SC-F344, PF-F344, PF-F344+Sita (sitagliptin) and PF-F344+Exe4 (exendin-4) groups. (b) In serum of wild-type rats, the correlation between the active GLP-1 levels and DPP4 activity was evaluated by Pearson correlation coefficients. (c) In serum of wild-type rats, the correlations between the active GLP-1 levels and the parameters of peritoneal transport, including D/D0 and D/S creatinine, were calculated. n = 6 for each group. Data represents mean ± SD; * indicates p-value< 0.05.

Supplementary 8. The uncropped Western blot images in main and supplementary figures.

Figure 1b

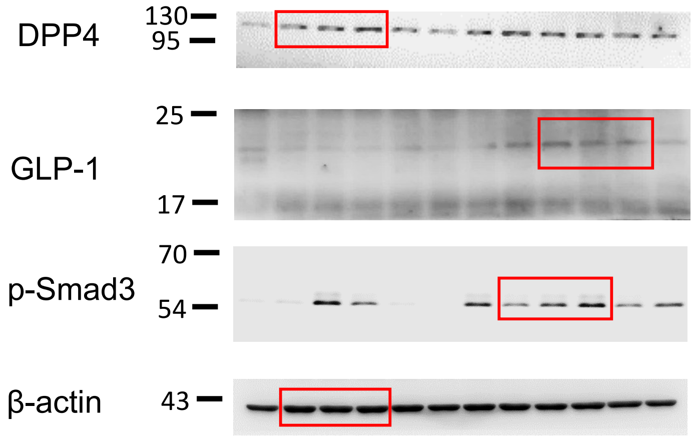

Figure 1c

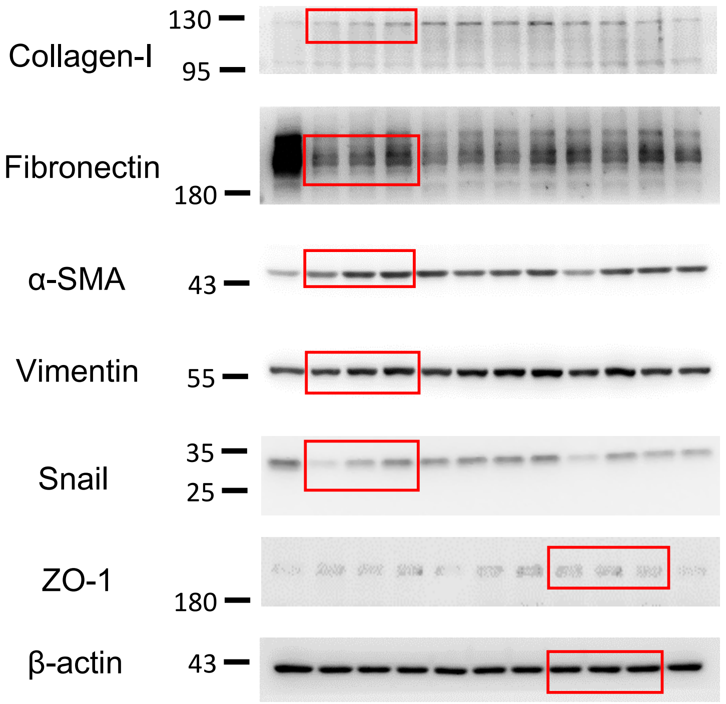

Figure 1d

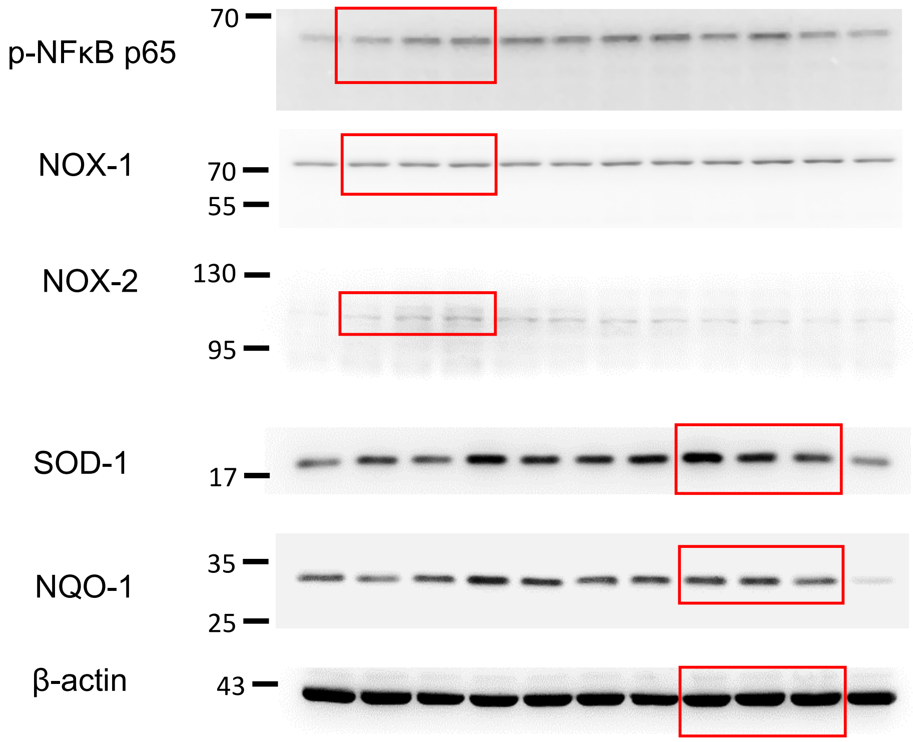

Figure 2b

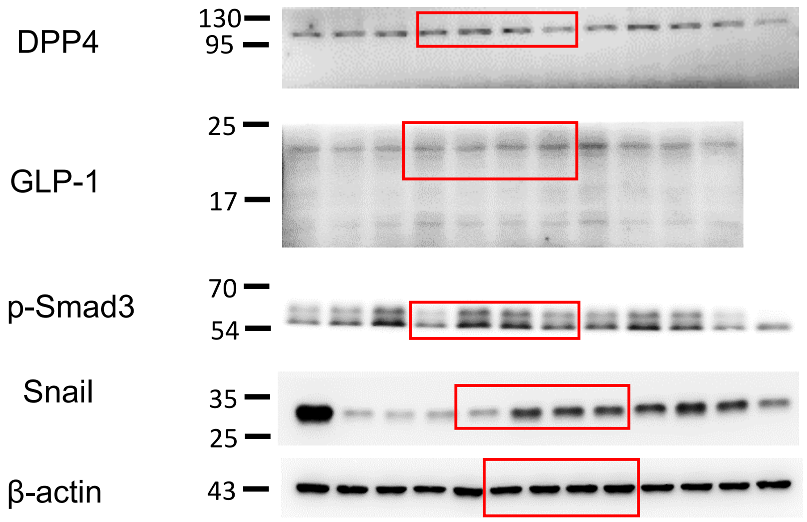

Figure 2c

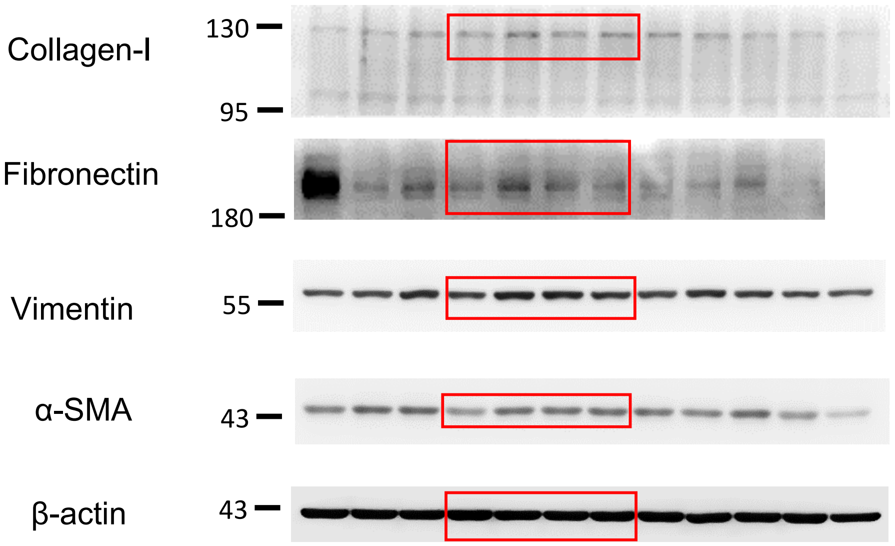

Figure 2d

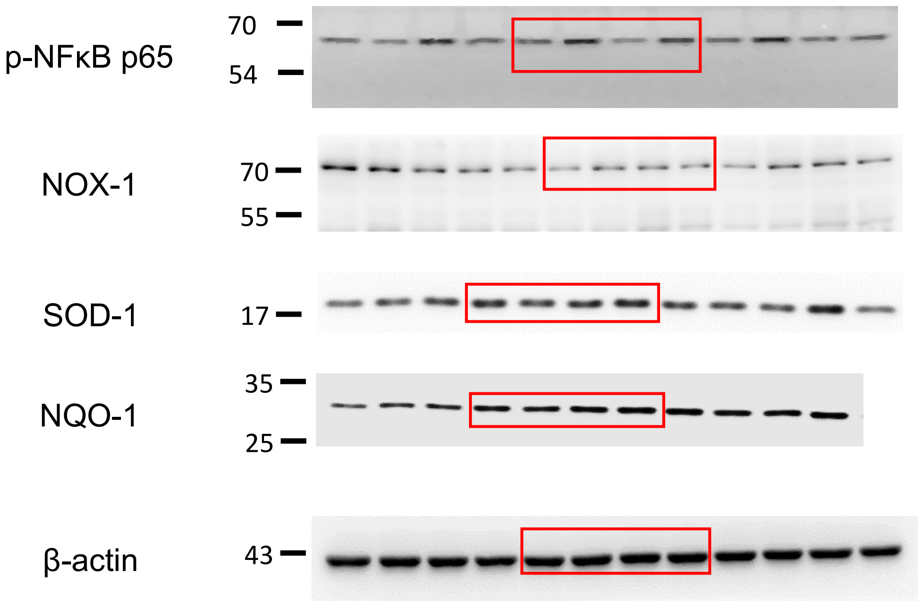

Figure 3b

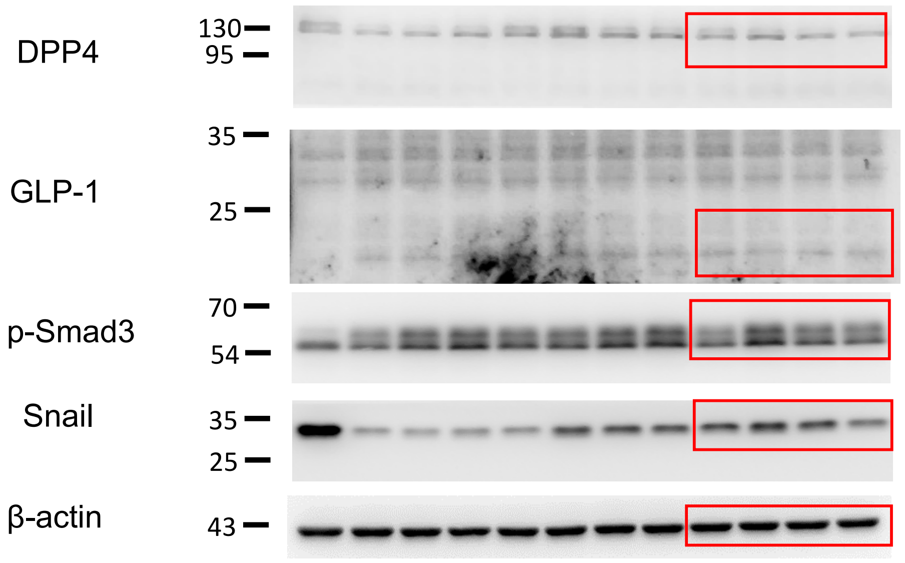

Figure 3c

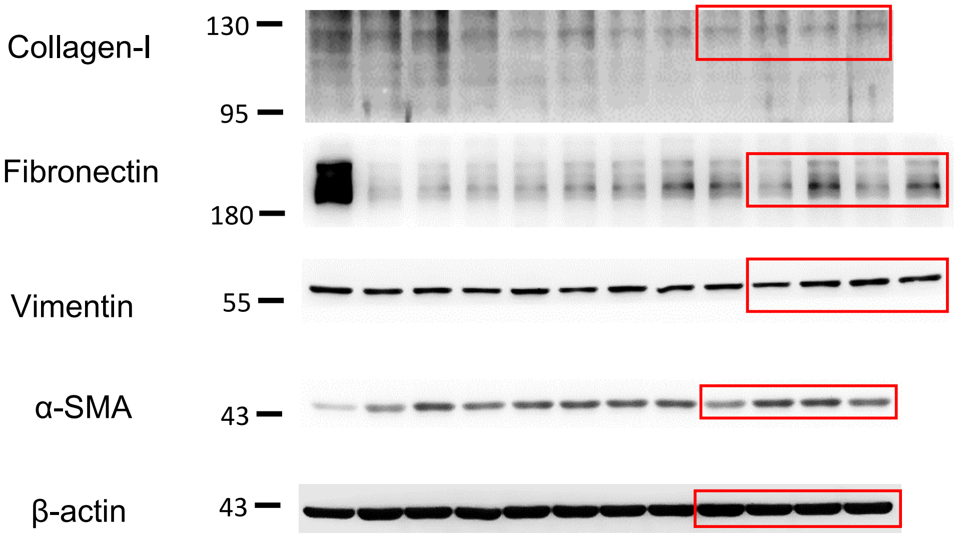

Figure 3d

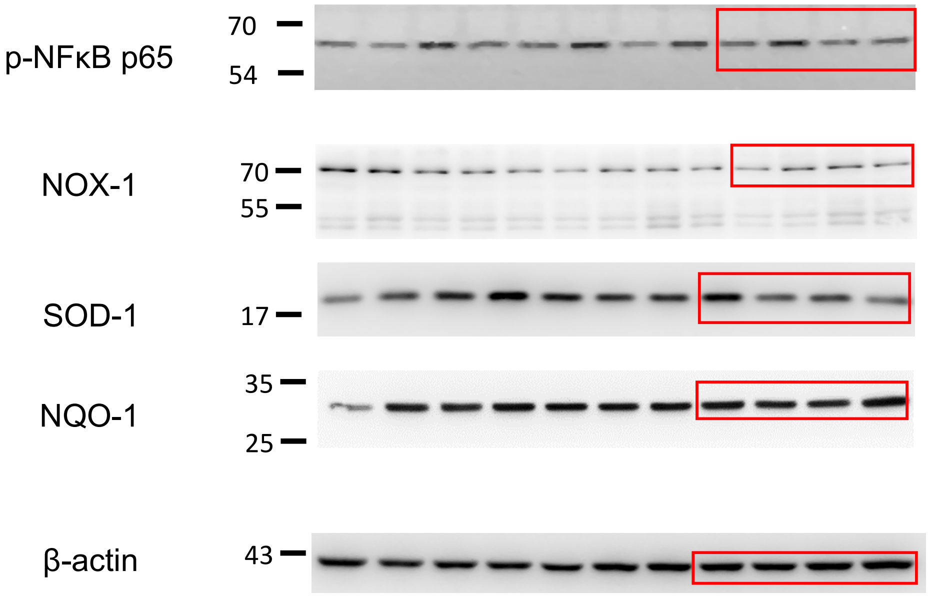

Figure 5d

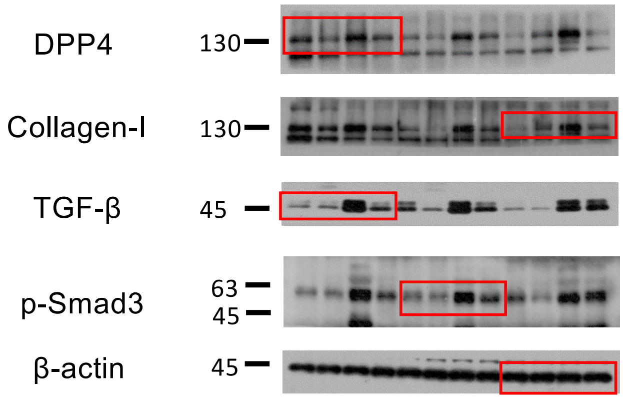

Figure 5e

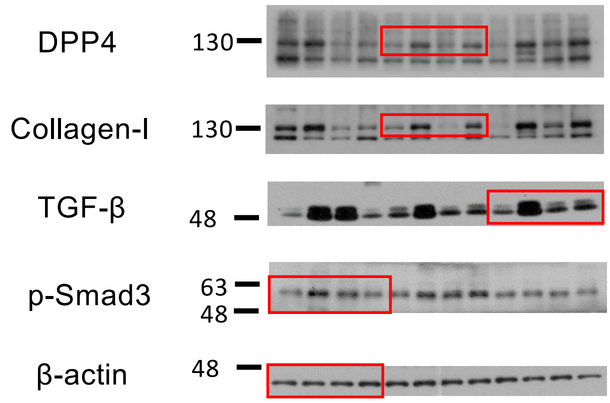

Figure 6g

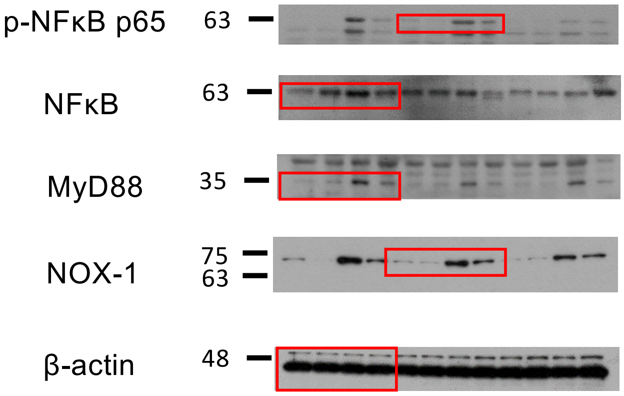

Figure 6h

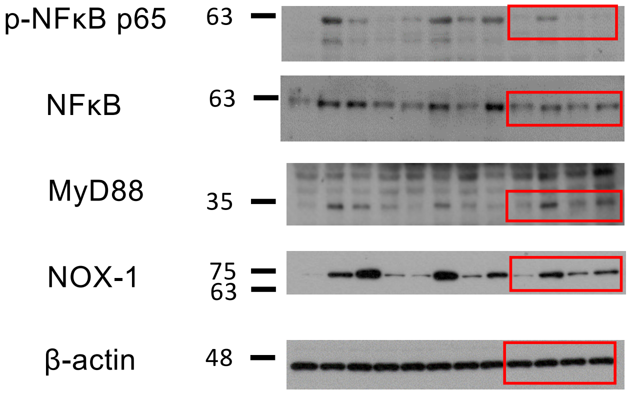

Supplymentery figure 1a

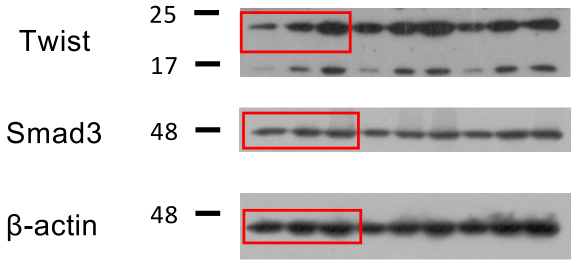

Supplymentery figure 1b

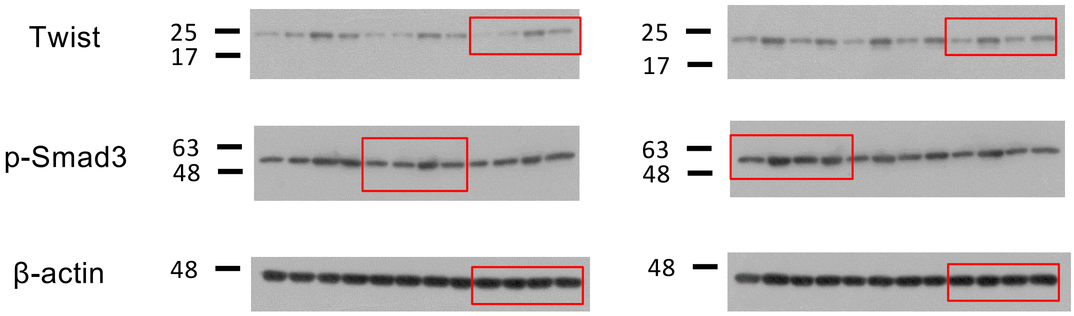

Supplymentery figure 2a-b

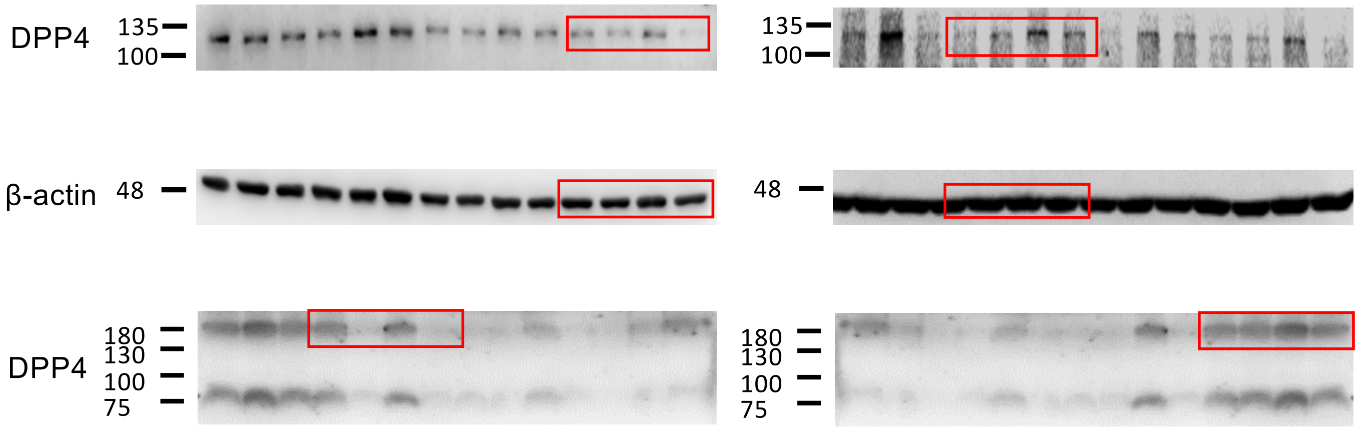

Supplymentery figure 4a

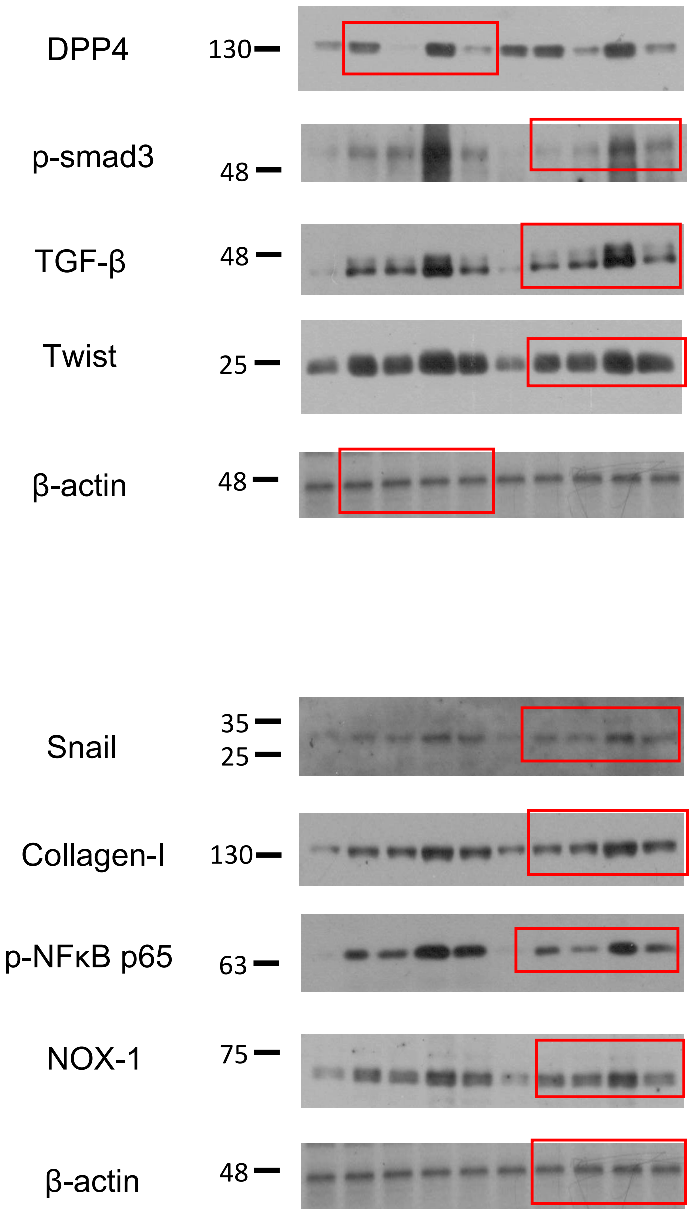

Supplymentery figure 6a

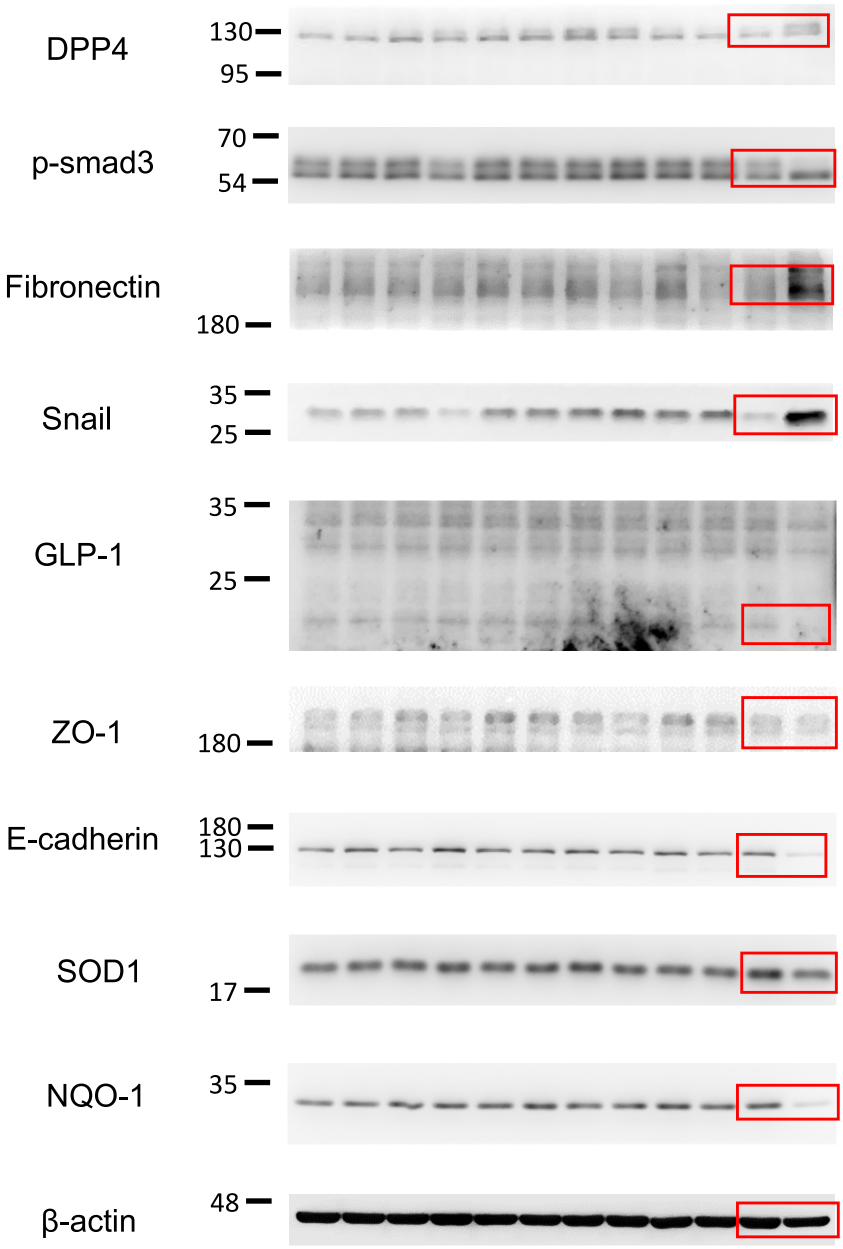

Supplementary Table

Supplementary Table 1. Antibody list.

Name	Dilute	Company	Cat No.	Application	Lot No.
GLP-1	1:1000	Abcam	ab22625	Western blotting	GR3178933-5
p-Smad3	1:1000	Abcam	ab28379	Western blotting	GR3194559-4
Smad3	1:1000	cell signaling	#9523	Western blotting	7
TGFβ	1:1000	Abcam	ab64715	Western blotting	GR3206406-1
collagen type I	1:1000	Abcam	ab34710	Western blotting	GR3192859-1
Fibronectin	1:1000	Abcam	Ab6328	Western blotting	GR3193980-1
α-SMA	1:1000	Sigma	A2547	Western blotting	077M4846V
Vimentin	1:1000	cell signaling	#5741	Western blotting	6
Snail	1:1000	cell signaling	#3879	Western blotting	12
ZO-1	1:500	Abcam	ab96594	Western blotting	GR287296-4
p-NFκB	1:1000	cell signaling	#3033	Western blotting	16
NFκB	1:1000	Abcam	ab16502	Western blotting	GR3220118-1
MyD88	1:1000	Abcam	ab2064	Western blotting	GR3183101-4
NOX1	1:1000	Sigma	SAB4200097	Western blotting	088M4789V
NOX2	1:1000	Sigma	SAB4200118	Western blotting	033M4873
SOD-1	1:1000	Abcam	ab16831	Western blotting	GR247287-3
NQO1	1:1000	Abcam	ab28947	Western blotting	GR232090-9
Twist	1:1000	Abcam	Ab49254	Western blotting	GR3282530-4
Actin	1:10000	Merck	MAB1501	Western blotting	3018859
DPP4	1:1000	Aviva Systems Biology	ARP63319	Western blotting, Immunofluorescence staining	QC34282-40883
DPP4	1:100	Thermo	MA5-13562	Immunofluorescence staining	TE2572308D
WT-1	1:1000	Abcam	Ab18.840	Immunofluorescence staining	GR3212808-1
vWF	1:200	Merck	Ab7356	Immunofluorescence staining	3139133
Alexa Fluor 488® phalloidin, F-actin probe	1:400	Invitrogen™	A12379	Immunofluorescence staining	2031929
collagen type I	1:1000	Abcam	Ab34710	Immunohistochemistry staining	GR3192859-1
CD45	1:100	Abcam	Ab10558	Immunohistochemistry staining	GR108450-1

Supplementary Methods

DPP4 siRNA transfection and TGF-β treatment

Met-5A cells were transfected with DPP4 siRNA (DPP4 Silencer Select®, Thermo Fisher Scientific) or negative control by Lipofectamine™ 3000 Transfection Reagent (#L3000-015, Invitrogen), followed by starvation and then 25 mM glucose exposure for 120 hr. On the other hand, Met-5A cells were treated 2 ng/ml TGF-β1 recombinant protein (240-B, R&D systems) for 120 hr to elucidate the molecular changes.

Immunocytochemistry staining

Met-5A cells were seeding in Millicell EZ SLIDE (PEZGS0416, Merck). After cells were exposed without glucose and with glucose w/o the inhibiting drugs, cells were fixed and then stained by DPP4 and phalloidin-labeled F-actin antibodies. All the antibody information and conditions were listed in supplementary Table 1 .

Assessment of circulating active GLP-1 level

Glucagon-Like Peptide-1 (Active) ELISA Kit (EGLP-35K, Millipore) was performed to detect active GLP-1 levels in circulation by using standard methods according to manufacturers' instructions.
