# Supplementary material for: Dipeptidyl peptidase 4 promotes peritoneal fibrosis and its inhibitions prevent failure of peritoneal dialysis
Source: Commun Biol. 2021 Jan 29;4:144. doi: 10.1038/s42003-021-01652-x (PMC7846859; doi:10.1038/s42003-021-01652-x)
Supplement: Supplementary file 2 — Description of additional supplementary files [file 42003_2021_1652_MOESM2_ESM.pdf]

Description of Additional Supplementary Files

File name: Supplementary Data 1
Description: Source data underlying plots shown in figures.
