# Supplementary material for: Dipeptidyl peptidase 4 promotes peritoneal fibrosis and its inhibitions prevent failure of peritoneal dialysis
Source: Commun Biol. 2021 Jan 29;4:144. doi: 10.1038/s42003-021-01652-x (PMC7846859; doi:10.1038/s42003-021-01652-x)
Supplement: Supplementary file 4 — Reporting Summary [file 42003_2021_1652_MOESM4_ESM.pdf]

nature research

Corresponding author(s): Chih-Chao Yang

Last updated by author(s): 2020/04/17

Reporting Summary

Nature Research wishes to improve the reproducibility of the work that we publish. This form provides structure for consistency and transparency in reporting. For further information on Nature Research policies, see Authors & Referees and the Editorial Policy Checklist .

Statistics

For all statistical analyses, confirm that the following items are present in the figure legend, table legend, main text, or Methods section.

n/a	Confirmed	
☐	☒	The exact sample size (*n*) for each experimental group/condition, given as a discrete number and unit of measurement
☐	☒	A statement on whether measurements were taken from distinct samples or whether the same sample was measured repeatedly
☐	☒	The statistical test(s) used AND whether they are one- or two-sided<br>*Only common tests should be described solely by name; describe more complex techniques in the Methods section.*
☐	☒	A description of all covariates tested
☒	☐	A description of any assumptions or corrections, such as tests of normality and adjustment for multiple comparisons
☐	☒	A full description of the statistical parameters including central tendency (e.g. means) or other basic estimates (e.g. regression coefficient) AND variation (e.g. standard deviation) or associated estimates of uncertainty (e.g. confidence intervals)
☒	☐	For null hypothesis testing, the test statistic (e.g. *F*, *t*, *r*) with confidence intervals, effect sizes, degrees of freedom and *P* value noted<br>*Give P values as exact values whenever suitable.*
☒	☐	For Bayesian analysis, information on the choice of priors and Markov chain Monte Carlo settings
☒	☐	For hierarchical and complex designs, identification of the appropriate level for tests and full reporting of outcomes
☒	☐	Estimates of effect sizes (e.g. Cohen's *d*, Pearson's *r*), indicating how they were calculated

Our web collection on statistics for biologists contains articles on many of the points above.

Software and code

Policy information about availability of computer code

Data collection	Excel
Data analysis	SPSS and Prism GraphPad

For manuscripts utilizing custom algorithms or software that are central to the research but not yet described in published literature, software must be made available to editors/reviewers. We strongly encourage code deposition in a community repository (e.g. GitHub). See the Nature Research guidelines for submitting code & software for further information.

Data

Policy information about availability of data

All manuscripts must include a data availability statement. This statement should provide the following information, where applicable:

- Accession codes, unique identifiers, or web links for publicly available datasets
- A list of figures that have associated raw data
- A description of any restrictions on data availability

The data was not available online because of government’s regulation and policy. However, data of interest could be requested for academic purpose after contacting corresponding author.

Field-specific reporting

Please select the one below that is the best fit for your research. If you are not sure, read the appropriate sections before making your selection.

☒ Life sciences ☐ Behavioural & social sciences ☐ Ecological, evolutionary & environmental sciences

Life sciences study design

All studies must disclose on these points even when the disclosure is negative.

Sample size	N = 174,686 for subjects diagnosed with type 2 diabetes mellitus (T2DM) and end-stage renal disease (ESRD).
Data exclusions	Those patients who have history of malignancy and liver cirrhosis, age of younger than 18 or older than 80 years, and follow-up period < 1 year were excluded from this study.
Replication	All data of diagnosis and drug prescription were retrieved from Taiwan National Health Insurance Research Database (NHIRD), reviewed and verified by Health Insurance Bureau, and can be applied for academic research.
Randomization	This is a retrospective nationwide population-based cohort study. Control group was selected after matching study group in a 1:10 ratio with age, gender, socioeconomic status, and relevant anti-diabetic medications.
Blinding	No blinding was used in this retrospective analysis.

Reporting for specific materials, systems and methods

We require information from authors about some types of materials, experimental systems and methods used in many studies. Here, indicate whether each material, system or method listed is relevant to your study. If you are not sure if a list item applies to your research, read the appropriate section before selecting a response.

Materials & experimental systems

n/a	Involved in the study	
☐	☒	Antibodies
☐	☒	Eukaryotic cell lines
☒	☐	Palaeontology
☐	☒	Animals and other organisms
☐	☒	Human research participants
☒	☐	Clinical data

Methods

n/a	Involved in the study	
☒	☐	ChIP-seq
☒	☐	Flow cytometry
☒	☐	MRI-based neuroimaging

Antibodies

Antibodies used	The information of antibodies was listed in Supplementary Table 1.
Validation	CD26 (ARP63319, Aviva Systems Biology) was the validated antibody by Antibodypedia. Other antibodies possessed relevant citations on the manufacturer's website.

Eukaryotic cell lines

Policy information about cell lines

Cell line source(s)	Mesothelial cells, Met-5A cells (65302, BCRC), was purchased from Bioresource Collection and Research Center in Taiwan.
Authentication	“The Bioresource Collection and Research Center (BCRC) is a nonprofit organization sponsored by the Taiwan government since 1982. The cell bank at BCRC has a strict quality control system for each banked cell line, including sterility, mycoplasma contamination tests, isoenzyme analysis, karyotyping analysis, and STR profiling analysis for human cells.”from In Vitro Cell Dev Biol Anim. 2013 Dec;49(10):743-5
Mycoplasma contamination	“The Bioresource Collection and Research Center (BCRC) is a nonprofit organization sponsored by the Taiwan government since 1982. The cell bank at BCRC has a strict quality control system for each banked cell line, including sterility, mycoplasma contamination tests, isoenzyme analysis, karyotyping analysis, and STR profiling analysis for human cells.”from In Vitro Cell Dev Biol Anim. 2013 Dec;49(10):743-5
Commonly misidentified lines (See ICLAC register)	We have checked the update version of Cross-Contaminations. Met-5A doesn’t exist in the list of misidentified cell line.

Animals and other organisms

Policy information about studies involving animals; ARRIVE guidelines recommended for reporting animal research

Laboratory animals	The pathogen-free, adult male, 8-10 weeks of age, wild-type Fischer 344 rats and DPP4 deficient rats were provided from Charles River Technology (BioLASCO, Taiwan) and utilized as study animals.
Wild animals	This study did not involve wild animals.
Field-collected samples	These animals were housed with 23–24°C controlled temperature and 12/12 hr light/dark cycles in animal center of our hospital, which was an animal facility approved by Association for Assessment and Accreditation of Laboratory Animal Care International (AAALAC; Frederick, MD, USA).
Ethics oversight	This animal procedure was approved by the Institute of Animal Care and Use Committee at Kaohsiung Chang Gung Memorial Hospital (Affidavit of Approval of Animal Use Protocol No. 2017111301) and performed according to the guidelines for the care and use of laboratory animals.

Note that full information on the approval of the study protocol must also be provided in the manuscript.

Human research participants

Policy information about studies involving human research participants

Population characteristics	Patients aged between 18 and 80 years with ESRD and T2DM (N = 174,686), including 19,828 patients with PD, 171,266 with HD and 6,787 with RT, were identified from 23.74 million Taiwanese residents.
Recruitment	Diagnosis was based upon ICD-9-CM codes and drug information was according to Anatomical Therapeutic Chemical (ATC) Classification System by World Health Organization (WHO). The drug needs to be prescribed in accordance with guidelines or regulations of Taiwan Health Insurance Bureau for payment. The correctness of retrieved data was endorsed by Taiwan Health Insurance Bureau.
Ethics oversight	The study design was approved by the Ethics Institutional Review Board of Chang Gung Memorial Hospital (No. 201702246B1).

Note that full information on the approval of the study protocol must also be provided in the manuscript.
